# Transitional chelal digit patterns in saprophagous astigmatan mites

**DOI:** 10.1007/s10493-024-00907-6

**Published:** 2024-04-15

**Authors:** Clive E. Bowman

**Affiliations:** Mathematical Institute, University of Oxford, Oxford, OX2 6GG UK

**Keywords:** Astigmata, Beehive, Dentition, Engineering, Functional ecomorphology, Mastication, Matrix comparisons, Morphometrics, Shape complexity

## Abstract

Changes in the functional shape of astigmatan mite moveable digit profiles are examined to test if *Tyrophagus putrescentiae* (Acaridae) is a trophic intermediate between a typical micro-saprophagous carpoglyphid (*Carpoglyphus lactis*) and a common macro-saprophagous glycyphagid (*Glycyphagus domesticus*). Digit tip elongation in these mites is decoupled from the basic physics of optimising moveable digit inertia. Investment in the basal ramus/coronoid process compared to that for the moveable digit mastication length varies with feeding style. A differentiated ascending ramus is indicated in *C. lactis* and in *T. putrescentiae* for different trophic reasons. Culturing affects relative investments in *C. lactis*. A markedly different style of feeding is inferred for the carpoglyphid. The micro-saprophagous acarid does not have an intermediate pattern of trophic functional form between the other two species. Mastication surface shape complexity confirms the acarid to be heterodontous. *T. putrescentiae* is a particularly variably formed species trophically. A plausible evolutionary path for the gradation of forms is illustrated. Digit form and strengthening to resist bending under occlusive loads is explored in detail. Extensions to the analytical approach are suggested to confirm the decoupling of moveable digit pattern from cheliceral and chelal adaptations. Caution is expressed when interpreting ordinations of multidimensional data in mites.

## Introduction

**Fig. 1:**
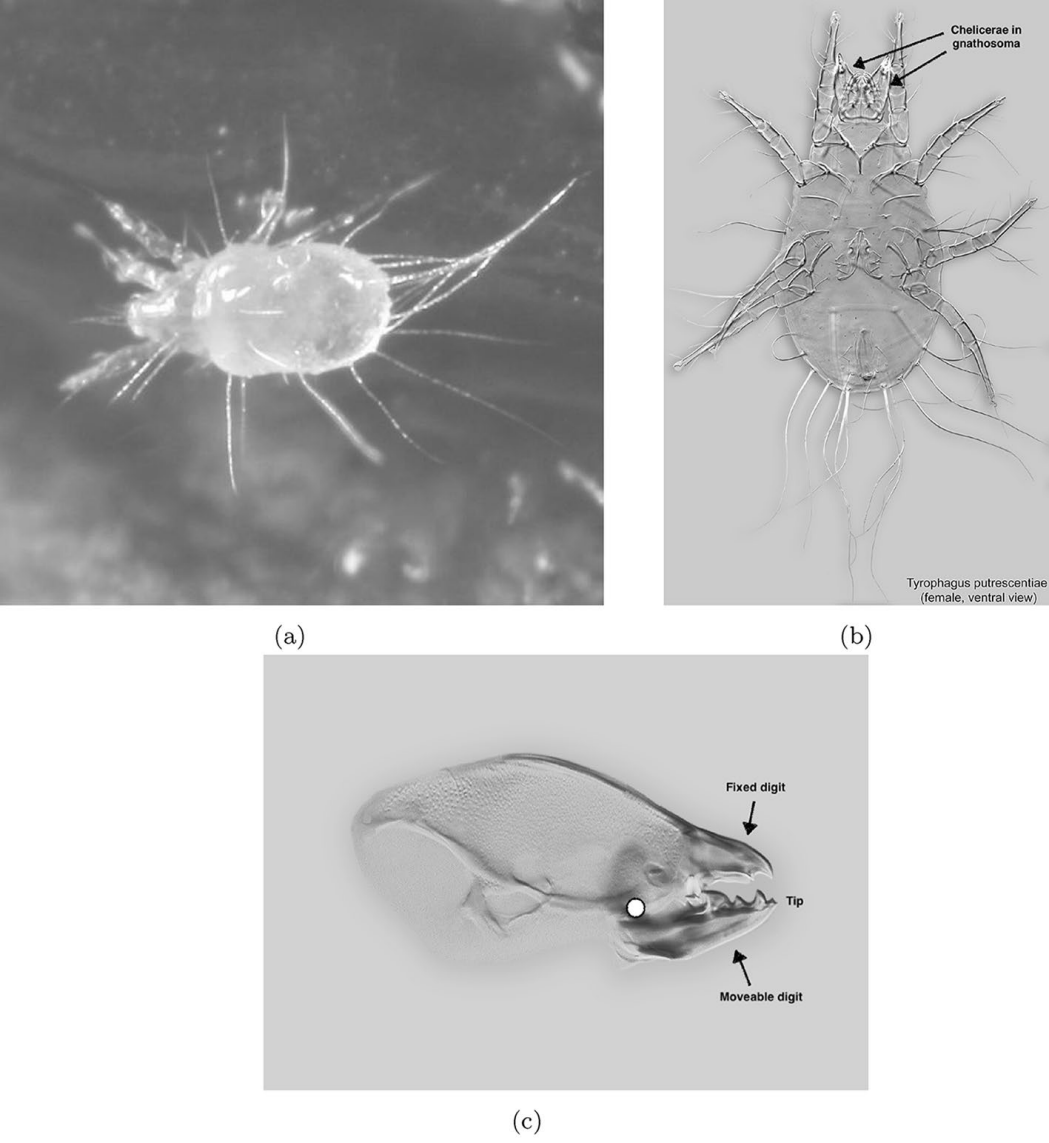
Example acarid astigmatan. Note these used to be called ‘astigmatids’. **a** Dorsal view of *Tyrophagus* sp. in a tortricid moth (probably *Episimus argutanus*) witch-hazel (*Hamamelis virginiana*) leaf-roll, Pelham, Hampshire County, Massachusetts, USA, August 3, 2013 ©2013 Charley Eiseman with permission. Gnathosoma to the left (partly hidden in klinorhynchid pose). **b** *Tyrophagus putrescentiae* female. Note birefringent cheliceral chelae anteriorly. Amended from a photo by Pavel Klimov, Bee Mite ID (idtools.org/id/mites/beemites) with permission. **c** Enlarged lateral view of a chelicera of *Chaetodactylus krombeini*. Note dentate chela to right end of cheliceral shaft. Tendons and musculature inside the cheliceral base actuate the (lower) moveable digit against the (upper) fixed digit (comprising the chela) around the articulating condyle (indicated by white circle). The gleaming actinochitinous nature of the digits points to their evolutionary origin from setae/ambulacra (Grandjean 1947). From colour photograph ex Pavel Klimov with permission

**Fig. 2:**
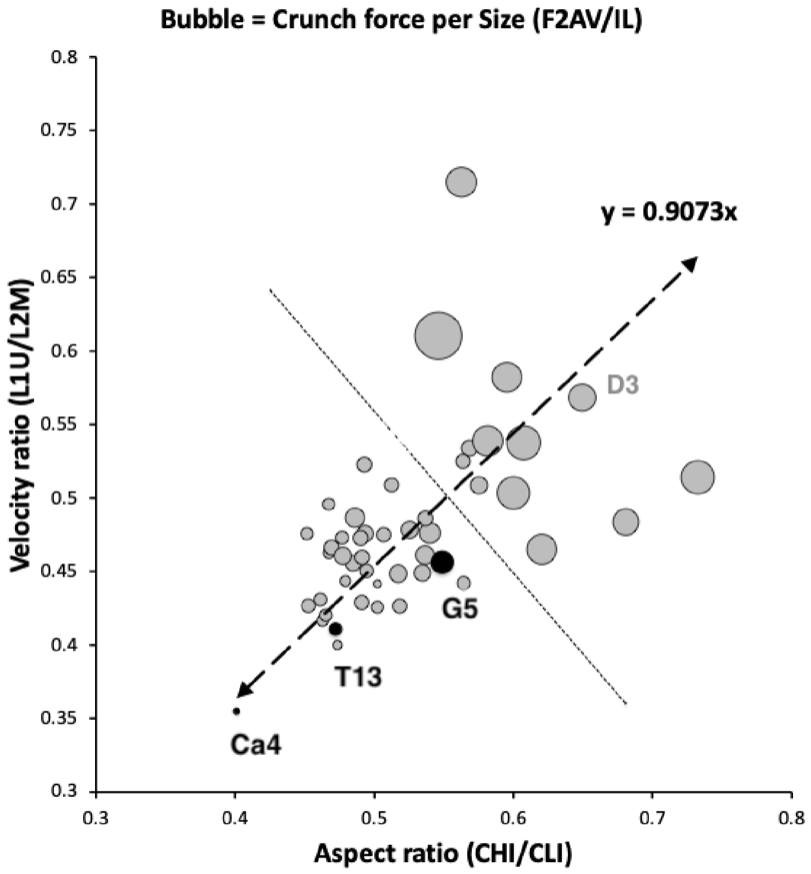
Summary plot from Bowman (2021b) with the three UK beehive species highlighted in lower functional ecomorphological group by black circles. Ca4 = *Carpoglyphus lactis*, T13 = *Tyrophagus putrescentiae*. G5 = *Glycyphagus domesticus*. Note the apparent inferred linear transition between them in terms of trophic design, when considering the interplay of cheliceral features (*x*-axis) and chelal features (*y*-axis). For population D3 (in grey) see Discussion. Abbreviations: L1U = closing input lever moment arm length, L2M = output lever moment arm length (reference axis), F2AV = adductive output moment lever arm ‘crunch’ force at tip of moveable digit on occlusion, IL = idiosomal index (measure of body size), CHI = cheliceral segment height, CLI = cheliceral length (aka ‘reach’)

Astigmatan mites (e.g., Fig. 1, previously referred to as ‘astigmatid mites’) are commensals of humans and insects, in some cases being serious pests (Hughes 1976). From Bowman (2021b), the two most extreme morphological forms (smallest to largest) in the general purpose saprophagous astigmatan trophic design (i.e., within the lower convex hull in Fig. 25 of that review) were the small *Carpoglyphus lactis* and the large *Glycyphagus domesticus*. *Tyrophagus putrescentiae* was an intermediate form somewhere in between in ordinations of cheliceral and chelal features (e.g., Fig. 2).

These three species co-occur in UK beehives (Bowman 2023) where they could feed upon various materials with their cheliceral chelae.

### Rationale for the study

The great naturalist Sir David Attenborough (in, Attenborough and the Great Sea Monster, BBC1 TV broadcast in the UK 1st January 2024) said “A skull can reveal more about an animal than any other part of it’s skeleton...it can tells us a great deal about how the animal lived”. This is true if you map the vertebrate head and jaws to a mite’s gnathosoma and chelicerae. Many useful conclusions about differential trophic adaptations are available from hominid studies (e.g., Laird et al. (2016)), however looking at just the sizes of chelal ornamentations (Bowman 2024) in mites is not the whole story in order to help explain any co-existence of apparently competitive species. Another issue arises. Not just to ask: “Do the sizes of individual chelal teeth (and gullets) match life-style?” but,

“Does the arrangement or *pattern* of dentition (i.e., the total set of asperities) vary between such mites (who are co-occurring in the same habitat) more than expected?”

As Bowman (2024) illustrates, each digit feature interacts with the others in an arrangement (or model type) to make up the particular operating characteristics of the chela. One is interested here in the overall *pattern*, not just the relative sizes of each moveable digit feature (as estimated by say division or regression of specific dental features with some sort of body size measure) on this composite masticatory tool.

Patterns over the mastication surface can be summarised by a correlogram i.e., the covariation of the height at each of their locations (along the *L*2*M* axis, i.e,. from articulating condyle to moveable digit tip). Bar the moveable digit tip and articulating condyle (Fig. 1), all of these co-ordinate locations are analogous ‘projections to a basis’ like semi-landmarks and are not strictly homologous locations as the latter are not known embryologically. What might a suitably posed covariance matrix of these features reveal? However, one does not want to lose the scale of acarine features, since nature sees the actual sizes of structures not just the scale of the variation about them. Care in the choice of summary measures is needed.

**Fig. 3:**
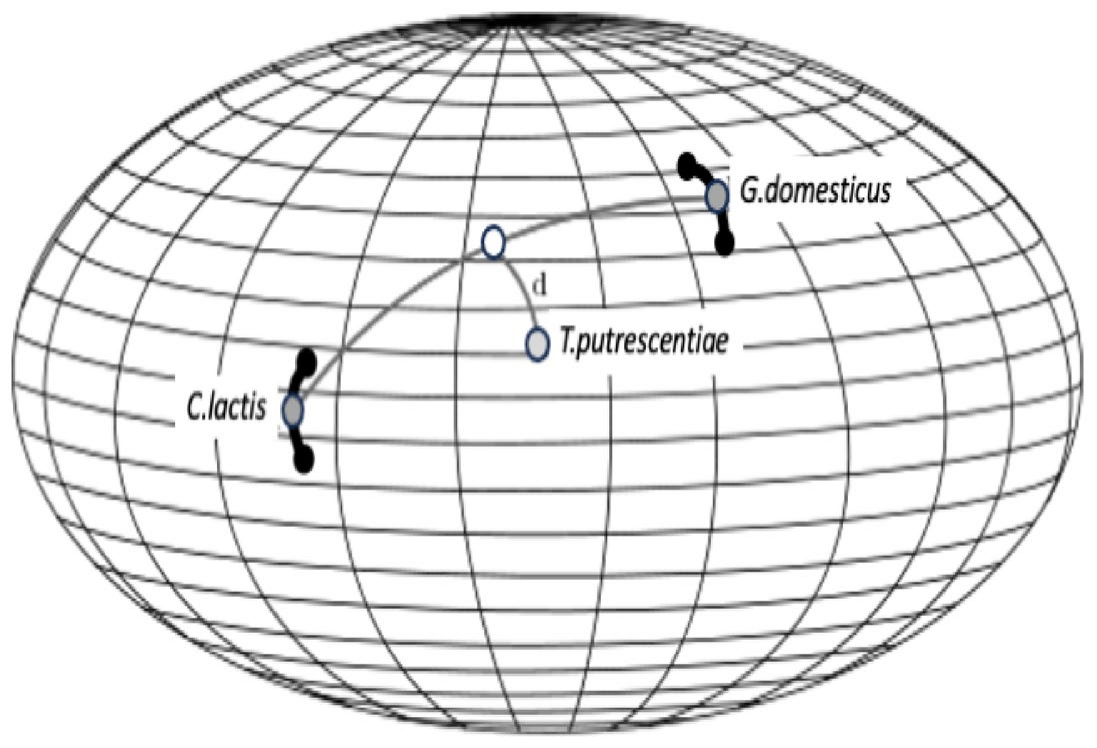
Geodesics (= minimum distance on a curved surface) in black between locations using an ellipsoid for simplicity of explanation. Dark grey dots = consensus $\varOmega$ covariance matrix between the wild-collected sample and laboratory sample of each reference taxon (*Carpoglyphus lactis* and *Glycyphagus domesticus*) as black dots connected by their own heavy black geodesic minimum path. Open dot = nearest matrix interpolate to a specified third $\varOmega$ ‘test’ covariance matrix for *Tyrophagus putrescentiae* (pale grey dot) with the minimum distance (*d*, labeled *lencheck*4 in the text herein for clarity of exposition) to the location from the geodesic between the former two reference taxa. Note *d* itself is along another geodesic. The true Riemannian manifold of symmetric positive definite matrices is cone-like. Amended from an illustration by oschenk June 2012 at https://math.stackexchange.com/questions/148839/what-is-the-geodesic-between-a-point-and-a-line-geodesic-between-two-points-on under ‘Fair use’

‘Common sense’ in a Euclidean space is that B is between A and C to the extent that distance(A,B)+distance(B,C) does not much exceed distance(A,C) however, symmetric positive definite matrices like covariance matrices are not Euclidean but exist on a smooth cone-like Riemannian metric surface or manifold (Fletcher and Joshi 2007; Bonnabel and Sepulchre 2009). What this non-linearity means is that morphing one matrix into another (e.g., evolutionarily changing from that digit profile pattern in *Carpoglyphus lactis* to say that in *Glycyphagus domesticus*, as extreme ‘reference taxa’) is not like a transit along a linear straight line on a piece of paper but is like moving from one position on a somehow curved surface to another (e.g., Fig. 3). Given this, is *Tyrophagus putrescentiae* a uniform transition between these two forms?

On a non-linear surface, the minimum distance trajectory is along a geodesic between the positions of moveable digit feature patterns, with the initial local movement from one position seemingly to appear to be going in the ’wrong’ direction when displayed on a flat map. Consider that if the curved surface that the covariance matrices were sitting upon was like that of the Earth. The shortest aeroplane flight from London to New York starts off in a North West direction and grazes the Arctic and is not straight across the Atlantic Ocean as on a planar map. A principal components analysis or singular value decomposition (SVD) of such matrices (for any one species) is like projecting where you are onto a flat plane tangentially resting at that location on the curved surface where you are (see Barachant et al. 2013). It has limited relevance to defining the non-linear transit between locations of covariance matrices unless the start and end of the journey are close nearby.

Fréchet smoothing by weighted Fréchet regression can define a plausible evolutionary path on a Riemannian metric between the two covariance matrices for *C. lactis* and *G. domesticus* as well as relating their location to other variables. Manipulation of weights allows one to step along that trajectory to find that place on the geodesic closest in terms of its Fréchet distance to the third covariance matrix for *T. putrescentiae* (Fig. 3). The fineness of this interpolation can be as desired for maximum accuracy of this line. Interpolations and extrapolations based upon the relationship of matrix location to other measured variables (like size given by idiosomal length index, or the moveable digit tip velocity ratio itself) can be made if required by Fréchet regression.

Given that one measures the chelal moveable digit profile carefully, one can critically investigate the relative investment in each part as to how this acarine tool performs.

## Materials and methods

Preserved slide material of independently determined female *Carpoglyphus lactis*, *Glycyphagus domesticus* and *Tyrophagus putrescentiae* was accessed by the author from a single beehive habitat, location and date in Redland, Avon, UK (totalling 52 female specimens). Details are given in Bowman (2023). Then twenty female *C. lactis*, *G. domesticus*, and *T. putrescentiae* preserved adults were examined from laboratory cultures (kept at the now defunct Pest Infestation Control Laboratory, London Road, Slough, UK) that had been maintained at 90% RH on a yeast and wheatgerm mix for many years in the 1970-1980s. Details are shown in Bowman (2021b).

Drawings of each mite and its chelicerae, (and for *T. putrescentiae* the lengths of its D1, D2 and L2 idiosomal setae) were made from all cleared mounted specimens using Nomarski interference phase-contrast microscopy with a drawing tube and micrometer scale. Idiosomal index (Lynch 1989) in $\mu$m was measured throughout and denoted *IL*. The lengths of dorsal setae (D1, D2, L2 - Griffiths et al. 1990) in $\mu$m were measured when necessary for *T. putrescentiae* in order to determine Don Griffiths’ likely breeding group using the classifier from Bowman (2021a). *T. putrescentiae* ‘B‘ is now assumed to be almost certainly the less commonly occurring close relative *Tyrophagus fanetzhangorum* (see Su et al. 2020) but definitive identification of voucher specimens already deposited in museums is awaited. *T. putrescentiae* ‘A‘ (the ‘commonly occurring form‘) retains its original name in this investigation following Klimov and OConnor (2009, 2010, 2015) (and is not renamed as *Tyrophagus communis* Fan and Zhang 2007). Laboratory individuals denoted ‘A/B‘ were those individuals when the classifier based upon setal lengths on one side of the mite disagreed with the conclusions for setal length on the other side. However, as mixed *Tyrophagus* sp. populations in a single long term culture was not expected these are considered as ‘A‘ for the analysis. Any individual scored as ‘A/B‘ in the wild-collected sample was excluded from the analysis as mixed populations in the ‘wild‘ may occur (Erban et al. 2016). Museum specimens were not used in this analysis.

**Fig. 4:**
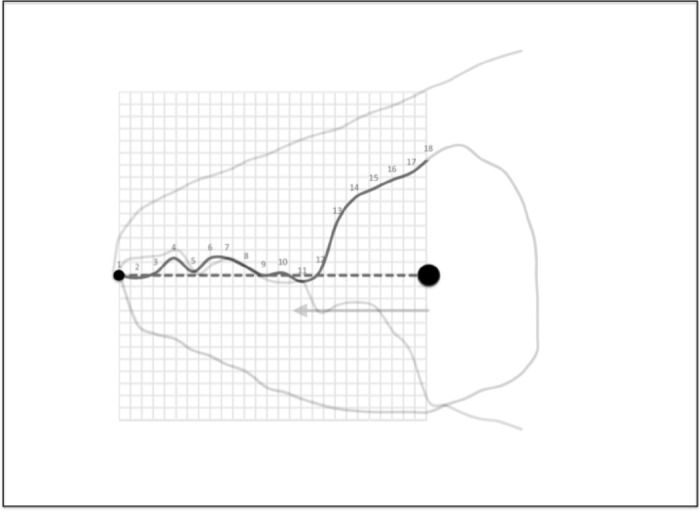
Measurement scheme for the three UK astigmatan mite beehive species illustrated with larger female *Tyrolichus casei* (Oudemans) chelicera for clarity, scaled to match standard grid overlay. Small black circle = moveable digit tip (landmark 1). Large black circle = condyle. Dashed line orientation to adductive moment lever arm (L2M). Heavy black line = moveable digit profile of projective locations (2–18). Large grey arrow = length of adductive lever moment arm *L*1*U* (see Bowman 2021b) forwards from condyle in *L*2*M* direction. Note tooth around projective location 4, gullet around projective location 5 and blade (projective locations 6ff) finishing approximately at an equivalent distance to *L*1*U* from the condyle (i.e., after projective location 11 where the moveable digit profile jerk is at a maximum; Bowman (2023)). Adductive tendon inserts on coronoid process illustrated maximum is at its maximum just posterior of projective location 18

Drawings were scanned using a HP OfficeJet Pro 8720 and digitised measurements of: idiosomal index length *IL*, setal lengths D1, D2 and L2; chelal design (adductive input lever moment arm length *L*1*U*, output lever moment arm length *L*2*M*, cheliceral height *CHI*, cheliceral length *CLI* following Bowman (2021b)) and cheliceral dentition [*x*, *y*] profiles with respect to the condyle-to-tip *L*2*M* axis were made using ImageJ 1.51s ex National Institutes of Health USA (http://imagej.nih.gov.uk/ij). Cheliceral abbreviations can be found in Fig. 2. Digit nomenclature and division into rami follows Fig. 5 in Bowman (2024). Mite chelae were orientated by reflection and rotation such that their adductive output lever moment arm directions (i.e., *L*2*M* see Bowman 2021b) were aligned. Two fixed homologous features (the moveable digit tip and the fixed digit to moveable digit articulating condyle) were used for registration (circles in Fig. 4) i.e., the *L*2*M* axis is the tribological reference line (Bhushan 2000). One landmark (the moveable digit tip), labeled (1) plus seventeen ‘projective locations’ (labeled 2-18) were determined by first scaling each *L*2*M* axis to the same size (rooted on the condyle) and then overlaying a equi-spaced 2D square grid in order to digitise the moveable digit profile (grey line in Fig. 4) at standard increments along the *L*2*M* axis from the tip. Projective location 18 was that directly vertical above the centre of the condyle seen laterally. This was not necessarily where the adductive tendon inserts into the ‘coronoid process‘ of the moveable digit (that is the length of $y_{18}$ after undoing the rescaling does not necessarily exactly match *L*1*U*). The moveable digit tip was taken to be the origin i.e., $[x=0,y=0]$.

Eighteen measurement points had been chosen to ensure potential full rank in any matrix decompositions of the laboratory-sourced sets of 20 specimens. The symbol ${\widehat{y}}$ is used to mean ‘estimated y’.

### Matrix analysis

Orientation to the *L*2*M* axis plus scaling of the digitising grid to be the same number of intervals from the moveable digit tip to the condyle (over mites of different moveable digit length) means that this *measurement* transformation is partially Procrustean i.e., the images have been reflected and rotated and scaled to a common notional register. No final reconfiguration dependent upon variance optimisation in location or scale was made. There are 17 data points (omitting the moveable digit tip at $x=0,y=0$) per individual. Only *y* is stochastic as the grid (i.e., $x_{1}...x_{18}$) is fixed by design. 2D geometric morphometrics is inappropriate.

Bowman (2024) introduces the concept of two different areas of possible moveable digit evolution. In one case, digit tip elongation is the main modality (together with dental ornamentation). In the other, a shape change in the ascending ramus/coronoid process is the modality.

Each region should be modelled independently. Accordingly data for the distal digit region was taken to be projective locations $2-13$, those for the proximal digit region projective locations $7-18$. The two numbers in the range of these projective locations are labelled as *upp* and *low*. Effectively this divides the *L*2*M* axis into two equi-sized pertinent regions (based on three morphological ‘windows’), allowing one to contrast the distal versus proximal shape changes (i.e., the horizontal ramus + ascending ramus versus the ascending ramus + basal ramus) yet ensuring a compensatory common transitional ‘knot’ (the ascending ramus) between them. As a cross-check of the validity of these windows, their overlapping common projective locations ($7-13$) neatly encompassed the range of all the actual locations of the end of the mastication surface ($x_{i{e}}$ Bowman 2023) for all the laboratory and all the wild-collected individuals of all three UK beehive species. Fortuitously, this also ensured full rank in any matrix manipulation for the wild-collected sample of *G. domesticus* where there were only 12 distinct individuals available.

Then, define the 12 x 1 column vector $$y_{i,j}=\left( \begin{array}{c} y_{i,j,low} \\ \vdots \\ y_{i,j,upp} \\ \end{array} \right)$$ for the *j*th individual of the *i*th taxon, where the third index is the appropriate projective location number. Assemble these columns over the $n_{i}$ individuals for that taxon by defining

$$\begin{aligned} Y_{i}=\left( \begin{array}{cccc} y_{i,1,low}&{}y_{i,2,low}&{}...&{}y_{i,n_{i},low} \\ \vdots &{} \vdots &{}...&{} \vdots \\ y_{i,1,upp}&{}y_{i,2,upp}&{}...&{}y_{i,n_{i},upp} \\ \end{array} \right) \end{aligned}$$

and calculate the symmetric matrix $\varOmega _{i}=\frac{1}{n_{i}}Y_{i}.Y_{i}^{T}$ (where ^T^ means transpose), which is a 12 x 12 average non-scaled non-centred SSCP matrix for the *i*th taxon. This is a positive semi-definite matrix (as for any vector $\beta \ne 0$, $\beta ^{T}.\varOmega _{i}.\beta \ge 0$). Its leading diagonal entries are $\ge 0$, none of its eigenvalues are negative and its determinant is non-negative. It can be thought of as a matrix analogue of a positive real number. As such it can be a convenient data summary to make overall comparisons for morphologists. It can be factorised by a Cholesky decomposition. In geometric terms, this condition is that, for every $\beta$, the angle between $\beta$ and $\varOmega _{i}.\beta$ does not exceed $\frac{\pi }{2}$.

Note that this is not a scaled corrected sums of squares and cross-products CSSCP matrix of normalised variates i.e., neither mean correction nor variance scaling was done. A pre-multiplier of $\frac{1}{n}$ was used for each matrix where $n=$ number of mites used for that matrix. The SSCP is explicitly labeled as ‘average’ here because *n* may vary from collection to collection. This approach retains the mean profile size for each taxon and allows larger variance measures to be deemed as more important in any comparisons (using the hypothesis test below).

So twelve average SSCP (*avSSCP*) matrices were formed: $\varOmega _{i,j,k}$ where

- *i* = [$$\textit{C. lactis, G. domesticus, T. putrescentiae}$$],
- $$j=['wild\ collected',\ laboratory]$$, and
- $$k=[distal,\ proximal]$$.

Note, distal = horizontal ramus + ascending ramus, proximal = ascending ramus + basal ramus.

Weighted (admixture) combinations of (*avSSCP*) matrices used ’estcov’ from the library ’shapes’ (Dryden 2021) to define the geodesic in the R version 4.3.1 (2023-06-16) – "Beagle Scouts" software system. The distance between (*avSSCP*) matrices used ’distcov’. Riemannian distances ( $||\ log[\varOmega _{a}^{-1/2} \varOmega _{b} \varOmega _{a}^{-1/2}]\ ||$ where $\varOmega _{a}$ and $\varOmega _{b}$ are the two matrices under consideration and ||....|| means ‘norm of’) were used throughout. This differentiable distance on the curved surface is affine i.e., invariant to a change in co-ordinate system. It’s geodesic for admixture *t* (of $t=0...1$) is defined by $\varOmega _{a}^{1/2}exp(t.log( \varOmega _{a}^{-1/2} \varOmega _{b} \varOmega _{a}^{-1/2})) \varOmega _{a}^{1/2}$ (Arsigny et al. 2006).

The observed (in Fig. 2 double arrow-headed dashed regression line) distinction between *C. lactis* and *G. domesticus* over the ‘ground space’ of cheliceral and chelal features was retained as the basis of the hypothesis test (Fig. 3).

### Overall hypothesis test

The over-arching hypothesis test for this study was:

“ Is the observed *pattern* of the moveable digit shape including its dentition in *Tyrophagus putrescentiae* (as represented by its *avSSCP*), an intermediate form or not between that observed for *Carpoglyphus lactis* and that observed for *Glycyphagus domesticus*? ”

This will be assessed with respect to the scale of variation between collections of the same species as follows.

By analogy with the test of whether a new data point is on a fitted linear regression line between two points (and thus needing to know the variation around that fitted line driven by the measurement errors at the two end points), the following was carried out for the proximal projective location dataset and separately for the distal projective location dataset (using Riemannian distances throughout):

- the geodesic distance (Fig. 3 thick black line) from $\varOmega$ for the wild-collected sample of *C. lactis* and its laboratory sample $\varOmega$ was estimated $\Rightarrow dClactis$
- the geodesic distance (Fig. 3 thick black line) from $\varOmega$ for the wild-collected sample of *G. domesticus* and its laboratory sample $\varOmega$ was estimated $\Rightarrow dGdomesticus$
- the average $\Rightarrow \ dsigma=0.5*(dClactis+dGdomesticus)$ was calculated, and taken to be the range of differences within a species
- the typical error $\sigma$ was set as $\approx \frac{range}{4}$ using the standard heuristic (‘range rule of thumb’ Wan et al. 2014), but then multiplied by 2 (as the distances herein are strictly positive). As a first approximation, the latter assumes that the variation that one would expect under the null for the three species are all similar
- the consensus $\varOmega$ for *C. lactis* was estimated as equivalent to an 1:1 equimixture of its wild-collected sample and laboratory sample matrices (along its geodesic, Fig. 3 grey circle)
- the consensus $\varOmega$ for *G. domesticus* was estimated as equivalent to an 1:1 equimixture of its wild-collected sample and laboratory sample matrices (along its geodesic, Fig. 3 grey circle)
- the length of the geodesic (Fig. 3 thin curved black line) between the consensus $\varOmega$ of *C. lactis* and the consensus $\varOmega$ for *G. domesticus* was estimated $\Rightarrow lengeodesic$.

Then by stepping along this *lengeodesic* (from the consensus $\varOmega$ matrix of *C. lactis* to the consensus $\varOmega$ matrix for *G. domesticus*) using differential weights (that still sum to 2) to form the interpolated estimated $\varOmega$ matrix at that point, the *minimum* distance (along its own ’new‘ geodesic, Fig. 3 open circle) from that point to the $\varOmega$ for the wild-collected *T. putrescentiae* (indicated by Fig. 3 pale grey circle) and separately from an equivalent (but different) interpolated $\varOmega$ point to the $\varOmega$ for the laboratory sample of *T. putrescentiae* (indicated by Fig. 3 pale grey circle) was found $\Rightarrow d$ in Fig. 3, (herein below labelled *lencheck*4 for clarity of exposition).

Given an assumption that small-scale random stochastic deviations from zero in geodesic distances could be considered locally as $\approx HalfNormal(0,\sigma ^{2})$, then the proximity (i.e., *lencheck*4) of a ‘test’ $\varOmega$ matrix (e.g., ’pale grey circle in Fig. 3) to the geodesic between the two reference taxa can be compared using a one-sided *z*-test to $\pm 1.645*\sigma$ of a *Normal*, and the ‘test’ $\varOmega$ marked as ‘not on the geodesic between the consensus $\varOmega$ of *C. lactis* and the consensus $\varOmega$ for *G. domesticus*’ if the former exceeds the latter. Any negative critical region is ignored. Think of this hypothesis test as seeing if the new sampled data point of *T. putrescentiae* is or is not within the expected ‘jitter’ of a fitted regression line where that variation is delivered by repeated samples of the two end-points. One could describe this process as a testing a fixed point against an estimated point in a ‘fat-line’ regression on the non-Euclidean surface (or conversely, comparing a single non-zero measurement from a sampled *Normal* distribution, to a null reference of zero). If the null is rejected, the chelal moveable digit *pattern* of *T. putrescentiae* is distinct from the nearest (and therefore any) smooth transformation between those *patterns* of the other two extremes. Note here that the ‘fatness’ of the critical zone does not diminish towards the midpoint of the regression, this width is driven by prediction errors assumed to be homoscedastic, not by the relative accuracy of line parameter estimates.

## Results

**Fig. 5:**
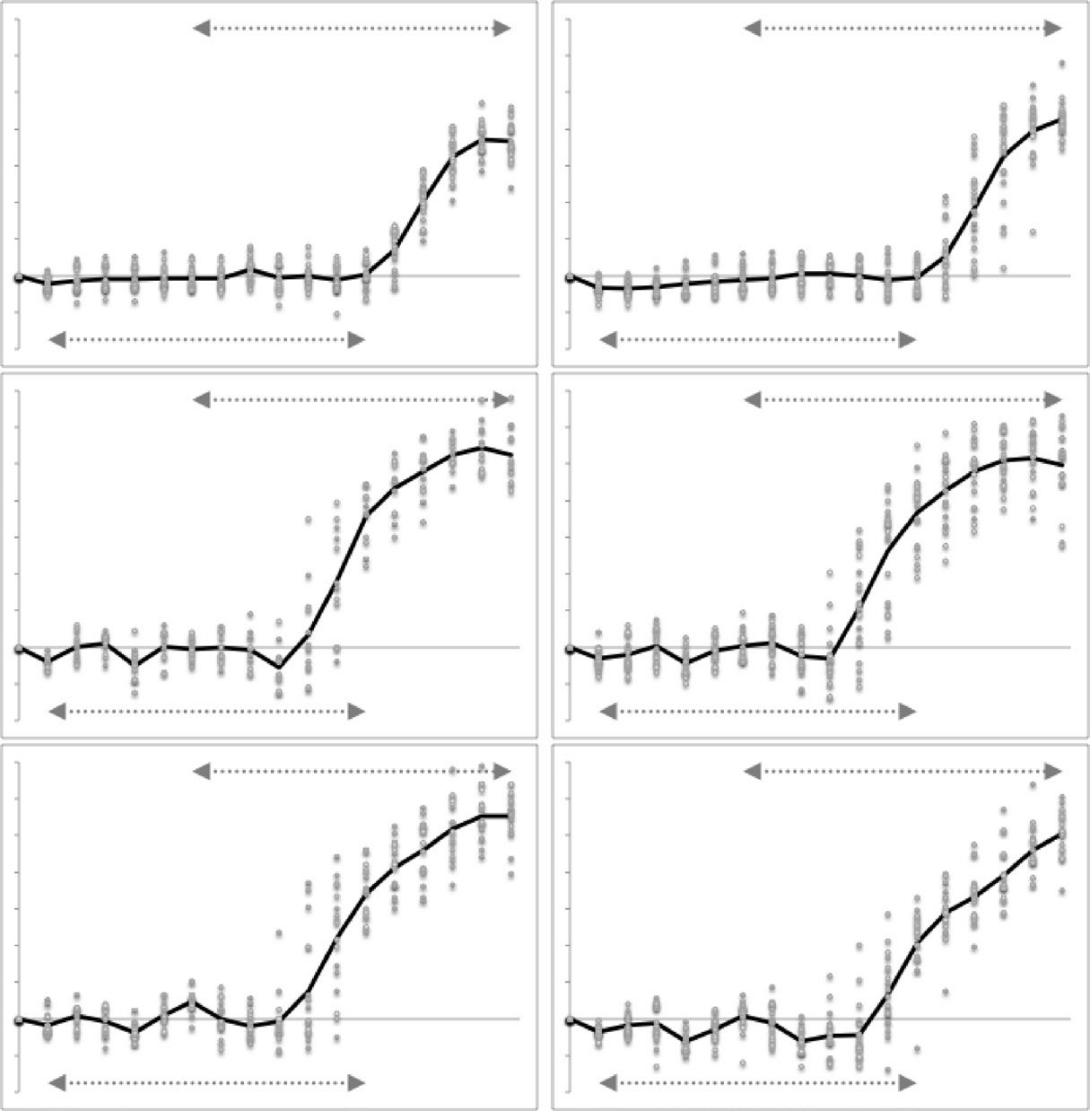
Observed moveable digit profiles (see Fig. 4) with their *L*2*M* axis scaled to match the nominal grid. Note differences in form. Upper double headed arrow = span of proximal projective locations. Lower double headed arrow = span of distal projective locations. Grey dots = individual measures. Black line = mean over individuals. Top row *Carpoglyphus lactis*,. Middle row *Glycyphagus domesticus*. Bottom row *Tyrophagus putrescentiae*. Left hand column = wild-collected sample (UK Beehive: *C. lactis* $n=21$, *G. domesticus* $n=12$, *T. putrescentiae* $n=17$). Right hand column = laboratory samples (Ca4, G5, T13, all $n=20$). Note induced higher vertical variation around some locations

Figure 5 illustrates the moveable digit profiles with their *L*2*M* axis scaled to match the nominal digitising grid. There was no evidence of distal differentially ‘set’ (Bowman 2024, Fig. 32) teeth or a comb-like ‘basket’ seen in algophagids like *Hericia* sp. by Fashing (2008) (or indeed as also found in pliosaurs) to capture slippery objects on the moveable digit of the studied carpoglyphid.

**Fig. 6:**
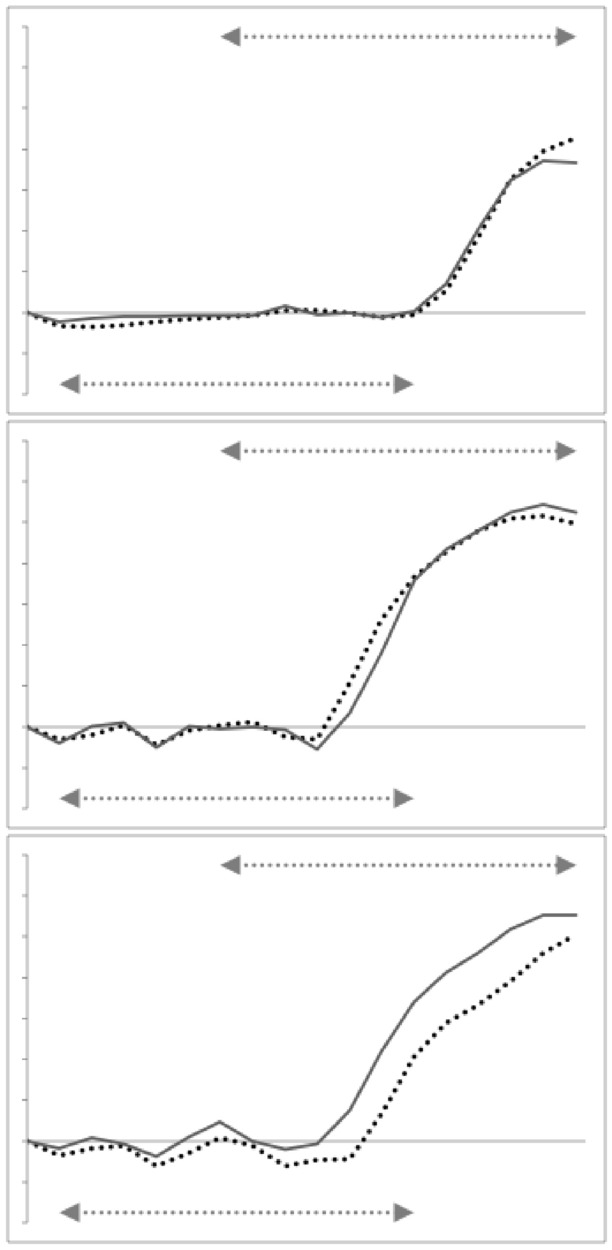
Observed mean moveable digit profiles (see Fig. 4) with their *L*2*M* axis scaled to match the nominal grid for each taxon. Note agreement across sample origin except in the acarid. Upper double headed arrow = span of proximal projective locations. Lower double headed arrow = span of distal projective locations. Black line = mean over wild-collected individuals. Dotted line = mean over laboratory sample. Top row *Carpoglyphus lactis*. Middle row *Glycyphagus domesticus*. Bottom row *Tyrophagus putrescentiae*

Note the good agreement of average profiles between the wild-collected and laboratory samples within *Carpoglyphus lactis* and within *Glycyphagus domesticus* (although the latter has a somewhat steeper ascending ramus in the laboratory culture sample). Note the marked deviation within *Tyrophagus putrescentiae* between its two samples, showing a proximal change in the location and shape of the ascending ramus and the basal ramus. Comparing the means (Fig. 6) shows that the basal ramus is proportionately diminished and both rami are more posterior in the laboratory sample (T13).

This does not infer that the *actual* mastication surface is longer. It is not, $m=15.4\ \mu$m in T13 (Bowman 2024), yet longer at $m=17.2\ \mu$m in the UK beehive sample (Bowman 2023) for *T. putrescentiae*. This is an overall shape change in the digit between samples for this species. Notwithstanding this, note the common patterns of dentition within each species across the two samples.

Indeed, averaging the two mean profiles within each species and visually comparing these shows that

**Fig. 7:**
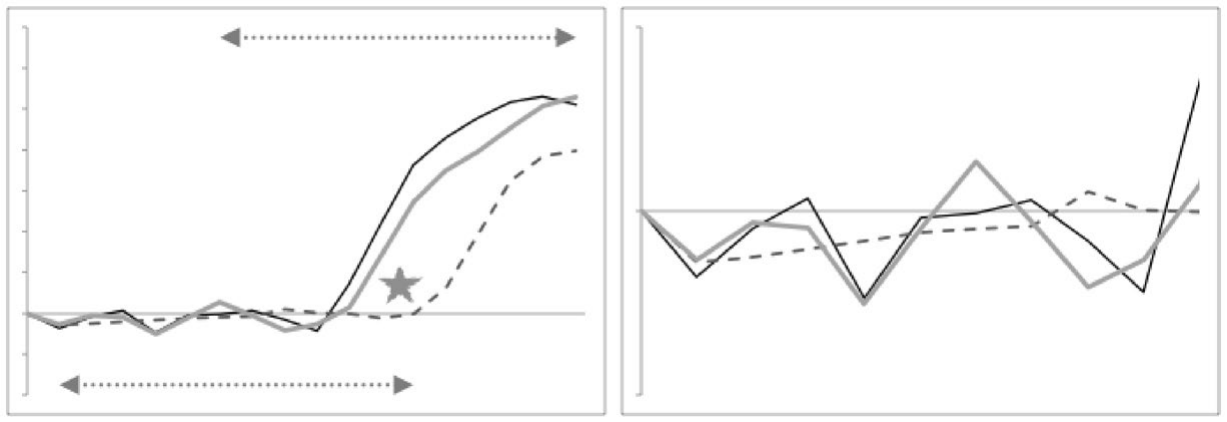
Averages over wild-collected and laboratory samples moveable digit profiles (see Fig. 4) with their *L*2*M* axis scaled to match the nominal grid for the three species. Upper double headed arrow = span of proximal projective locations. Lower double headed arrow = span of distal projective locations. Black line = *Glycyphagus domesticus*. Dotted line = *Tyrophagus putrescentiae*. Dashed line = *Carpoglyphus lactis*, Left panel at full-scale. Grey star shows different relative location for ascending ramus between species. Right panel zoomed in (by 2.6 times) to show mastication surface region. Note two equal height asperities for the saw-like profile of the glycyphagid, the unequal height for the two peaks in the acarid showing its central tooth, and the distinctly different depressed blade plus one small proximal tooth (of three) in the carpoglyphid

- the location of the ascending ramus varies between the two reference consensus species (Fig. 7 left)
- the shape of the ‘ascending ramus+basal ramus’ assembly of *T. putrescentiae* sits *somewhere* between the extremes of *C. lactis* and *G. domesticus* (Fig. 7 left, but whether this is a uniform transition is not clear).
- from the moveable digit tip posteriorly, the [dip, rise, dip, rise, dip] pattern of the horizontal ramus mastication surface is common across the glycyphagid and acarid with *C. lactis* being distally ‘bar-like’ (Fig. 7 left).
- *T. putrescentiae* has a clear central tooth of extra height (Fig. 7 right) compared to the ‘saw-like’ pattern of similar height asperities in *G. domesticus* as found by Bowman (2024).
- there is some evidence of the proximal small teeth region in *C. lactis* even at this scale of digitising.

### Relative investment between digit rami

**Table 1:** Extra measures by wild-collected specimen and UK astigmatan beehive species

Specimen	*nbO*[*VR*]	*F*1	$m_{ar}$	$\widehat{E_{hr}}$	$\widehat{E_{ar}}$	${\hat{E}}$ ratio
*Carpoglyphus lactis*						
224(1)-1	1.478	992.8	14.4	66.8	21812.6	326.3
224(1)-5	1.319	1050.0	16.9	30.7	34553.4	1124.7
224(1)-6	1.133	1008.3	17.8	33.9	20873.7	615.7
224(1)-6a	0.944	1031.3	18.8	21.4	17737.9	830.5
224(1)-6b	1.167	1071.6	15.5	21.5	11288.3	524.0
224(1)-6c	1.140	808.7	18.0	19.1	18747.2	981.3
224(1)-10	1.417	1105.7	15.9	50.3	25486.1	507.1
224(1)-11	1.109	888.2	19.1	188.0	14353.3	76.4
224(1)-11a	1.363	1039.8	16.4	95.8	15613.1	163.0
224(1)-11b	1.188	1096.5	15.9	38.7	16224.4	418.7
224(1)-11c	1.198	934.3	19.1	131.1	30426.8	232.1
224(1)-12	1.151	934.3	18.9	156.6	27730.7	177.1
224(1)-12a	1.076	1198.0	20.9	15.0	36406.4	2421.3
224(1)-13	0.968	962.4	15.9	152.0	5827.2	38.3
224(1)-13a	0.910	995.1	18.8	6.4	19610.4	3086.0
224(1)-15	1.032	894.1	18.7	59.0	14371.9	243.8
224(1)-15a	0.946	1103.9	16.5	18.3	28708.5	1567.9
224(1)-15b	1.103	1112.9	19.9	218.3	33631.9	154.0
224(1)-16	1.096	902.3	17.8	622.0	28964.6	46.6
224(1)-18	1.253	1240.8	18.4	46.2	163130.4	3529.4
224(1)-19	1.099	854.7	16.0	1306.9	38526.8	29.5
Summary	1.147	1010.8	17.6	157.0	29715.5	814.0
SD	0.153	111.8	1.7	296.0	31822.7	1017.4
*Glycyphagus domesticus*						
224(2)-1	1.045	3080.4	29.9	482.6	133633.4	276.9
224(2)-2	1.087	2596.8	27.8	1203.2	630653.4	524.1
224(2)-3	1.030	2926.0	30.9	591.6	152658.3	258.0
224(2)-4	1.113	3030.2	24.5	683.2	126924.2	185.8
224(2)-5	0.927	2362.8	26.6	1096.7	230415.1	210.1
224(2)-6	1.399	2825.5	25.0	138.4	114780.3	829.2
224(2)-7	1.201	2508.7	22.6	459.6	271750.7	591.3
224(2)-8	1.168	2605.9	23.0	59.2	136229.4	2300.5
224(2)-9	1.018	2482.1	32.2	1511.8	189124.8	125.1
224(2)-9a	1.124	2736.8	26.8	333.1	150969.0	453.2
224(1)-10	1.044	2202.2	25.0	1117.5	197204.9	176.5
224(1)-20	1.087	2458.4	25.1	367.6	146807.1	399.4
Summary	1.104	2651.3	26.6	670.4	206762.6	527.5
SD	0.118	272.5	3.1	459.4	141312.5	595.3
*Tyrophagus putrescentiae*						
224(1)-2	1.364	1575.0	16.2	17.3	12638.9	729.9
224(1)-3	1.107	2103.9	25.2	667.8	942097.8	1410.7
224(1)-4	1.145	2191.0	24.3	317.5	247726.7	780.3
224(1)-5a	1.029	2370.1	29.3	298.2	341908.9	1146.6
224(1)-6	1.110	1814.8	25.3	61.1	79532.7	1302.7
224(1)-6a	1.115	2651.8	25.4	349.1	293384.6	840.3
224(1)-7	1.172	2209.0	21.4	247.1	131184.9	530.8
224(1)-7a	1.256	1819.1	20.9	473.9	366689.2	773.7
224(1)-7b	1.045	2254.6	29.5	110.2	249114.9	2261.6
224(1)-7c	1.138	1691.3	24.5	303.6	161392.3	531.5
224(1)-7d	1.057	2232.9	26.3	453.0	343540.5	758.4
224(1)-8a	1.215	2296.9	24.7	62.6	632882.3	10111.8
224(1)-8b	1.044	1906.6	32.0	285.9	170223.4	595.4
224(1)-10	1.202	1459.3	19.7	52.6	145109.5	2759.6
224(1)-10a	1.244	1745.5	20.4	31.8	113069.5	3557.8
224(1)-14	0.946	2187.5	31.8	227.2	489427.0	2154.6
224(1)-odd2	1.209	1962.9	21.7	40.9	48988.5	1196.8
Summary	1.141	2027.8	24.6	235.3	280524.2	1849.6
SD	0.102	315.5	4.3	186.8	235010.0	2298.1

Summary is Mean and standard deviation (SD). *nbO*[*VR*] = average $VR'$ for ‘no bite’ moveable digit surface ( = ascending ramus after end of mastication surface at $x_{i_{e}}$ over the coronoid process to projective location 18 inclusive). *F*1 = adductive tendon force. $m_{ar}$ = ‘drape distance’ for ascending ramus i.e., after end of mastication surface (at $x_{i_{e}}$) over the coronoid process to projective location 18 inclusive. $\widehat{E_{hr}}$ = Estimated (scaled) Young’s modulus for horizontal ramus. $\widehat{E_{ar}}$ = Estimated (scaled) Young’s modulus for ascending ramus. ${\hat{E}}$ ratio $= \frac{E_{ar}}{E_{hr}}$

**Table 2:** Average $VR'$ for ‘no bite’ moveable digit surface *nbO*[*VR*] = ascending ramus after end of mastication surface (at $x_{i_{e}}$) over the coronoid process to projective location 18 inclusive and adductive tendon force (*F*1) by laboratory specimen and UK astigmatan beehive species

Specimen	*nbO*[*VR*]	*F*1
*Carpoglyphus lactis*		
Ca4-1	1.260	833.2
Ca4-2	1.306	946.2
Ca4-3	1.229	767.6
Ca4-4	1.376	956.9
Ca4-5	1.253	713.4
Ca4-6	1.321	759.3
Ca4-7	1.187	884.6
Ca4-8	1.433	952.0
Ca4-9	1.217	648.5
Ca4-10	0.958	703.6
Ca4-11	1.113	929.0
Ca4-12	1.949	774.5
Ca4-13	1.375	996.5
Ca4-14	1.189	945.0
Ca4-15	1.223	762.5
Ca4-16	1.203	812.2
Ca4-17	2.130	769.1
Ca4-18	1.420	1096.5
Ca4-19	1.012	740.2
Ca4-20	1.151	937.7
Summary	1.315	846.4
SD	0.277	118.5
*Glycyphagus domesticus*		
G5-1	1.319	2324.2
G5-2	1.048	2513.2
G5-3	1.050	2774.3
G5-4	1.089	2217.5
G5-5	1.127	2245.1
G5-6	1.205	2397.6
G5-7	1.140	2135.6
G5-8	1.246	2475.7
G5-9	0.989	2134.6
G5-10	1.097	2341.9
G5-11	1.034	2681.8
G5-12	1.176	3306.0
G5-13	1.008	2165.5
G5-14	1.056	2862.1
G5-15	1.102	2246.8
G5-16	1.056	1948.4
G5-17	1.130	2134.7
G5-18	1.107	2029.1
G5-19	1.024	2067.4
G5-20	1.014	2419.0
Summary	1.101	2371.0
SD	0.085	329.6
*Tyrophagus putrescentiae*		
T13-1	1.209	1099.3
T13-2	1.187	1252.1
T13-3	1.372	2073.2
T13-4	1.121	1285.0
T13-5	1.481	1349.6
T13-6	1.355	1444.4
T13-7	1.102	1292.1
T13-8	1.173	1491.4
T13-9	1.310	1474.9
T13-10	1.398	1594.1
T13-11	1.232	1459.7
T13-12	1.187	1252.1
T13-13	1.312	1344.8
T13-14	1.276	1440.5
T13-15	1.024	1373.2
T13-16	1.232	1404.7
T13-17	0.921	1387.4
T13-18	1.273	1378.0
T13-19	1.316	1348.8
T13-20	2.130	1346.1
Summary	1.280	1404.6
SD	0.239	189.6

**Fig. 8:**
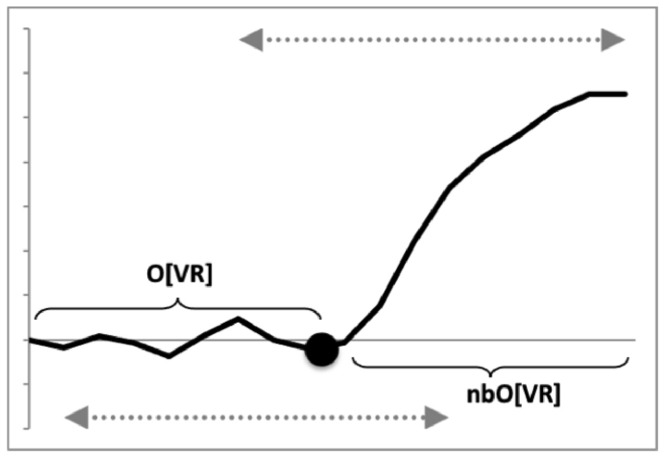
A typical wild-collected moveable digit profile (see Fig. 4) for *Tyrophagus putrescentiae* showing disjoint velocity ratio summary measures used herein. *O*[*VR*] = Observed average velocity ratio over mastication surface. *nbO*[*VR*] = Observed average velocity ratio of moveable digit profile posterior of mastication surface. Black dot is the end of mastication surface at $x=x_{i_{e}}$. Upper double headed arrow = span of proximal projective locations. Lower double headed arrow = span of distal projective locations

Bowman (2024) outlines in detail the observed average velocity ratio summary *O*[*VR*] as a measure of the overall practical utility of a moveable digit mastication surface (i.e., the horizontal ramus, $x \le x_{i_{e}}$) for food trituration allowing for changes in condyle position etc. This calculation (based upon $VR'$ for each asperity) can also be carried out for the comparative shape of the moveable digit posterior of this (i.e., at $x>x_{i_{e}}$), even though the surface is clearly ‘not biting’. This yields a measure *nbO*[*VR*] shown in Fig. 8 with results in Tables 1-2.

**Fig. 9:**
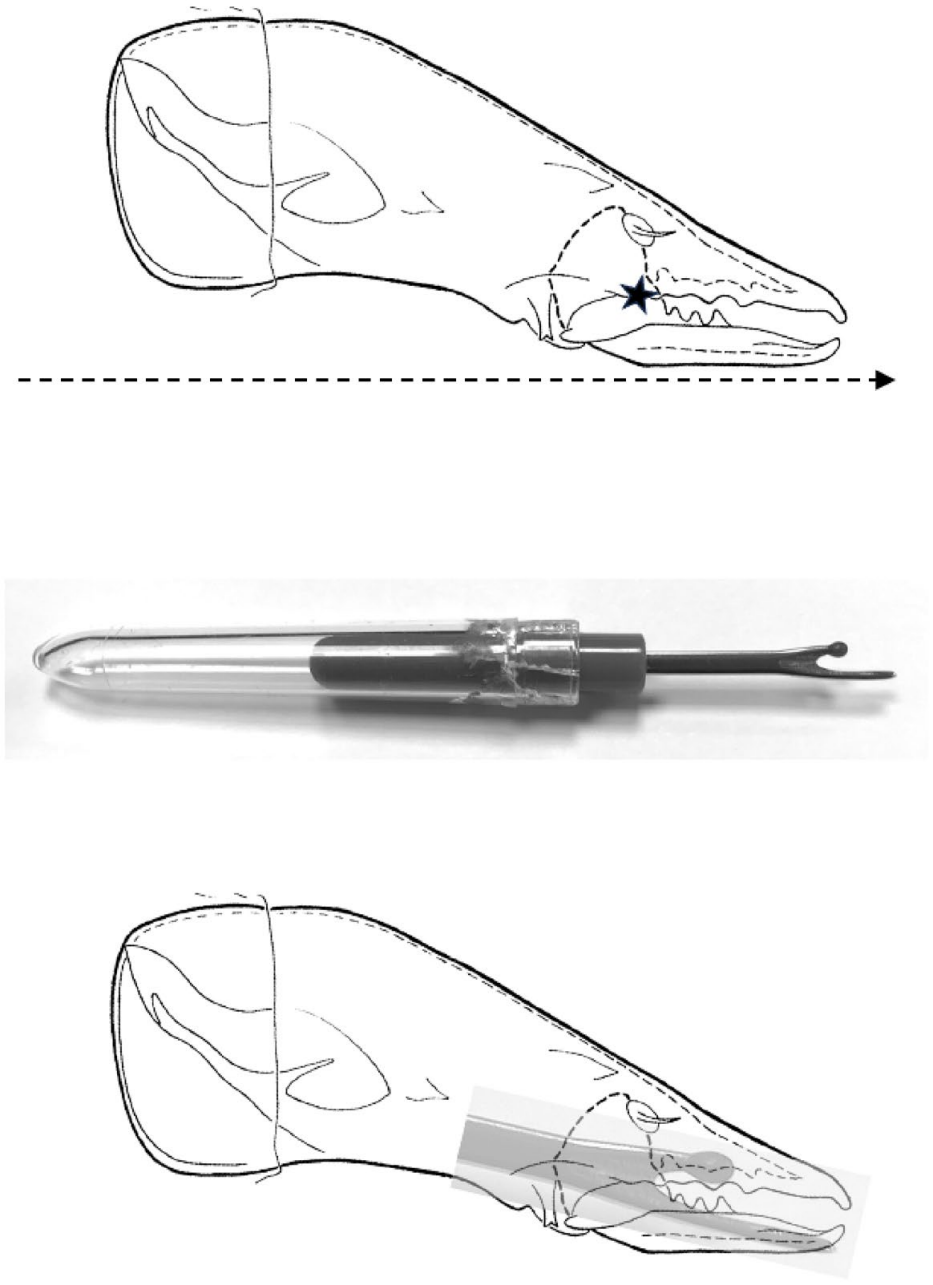
Skimming chela of *Carpoglyphus lactis* may slice through material like a tailor’s ‘stitch unpicker’. Upper, chelicera moves forward (dashed arrow). Star = ascending ramus as extra cutting edge on cheliceral protrusion. Middle, tailor’s stitch unpicker. Lower, stitch unpicker overlain on chela. Note how the sharp axil of the unpicker (that slices through threads) matches the mite’s ascending ramus when the two blades are aligned. Consiliently the bulging tip of the unpicker exactly matches the relative location of the three small digit teeth along the general axis, suggesting a common holding function. Mite picture from Johnston (1965) with personal permission and under ‘Fair use’ (https://guides.osu.edu/copyright/copyright-exceptions)

**Fig. 10:**
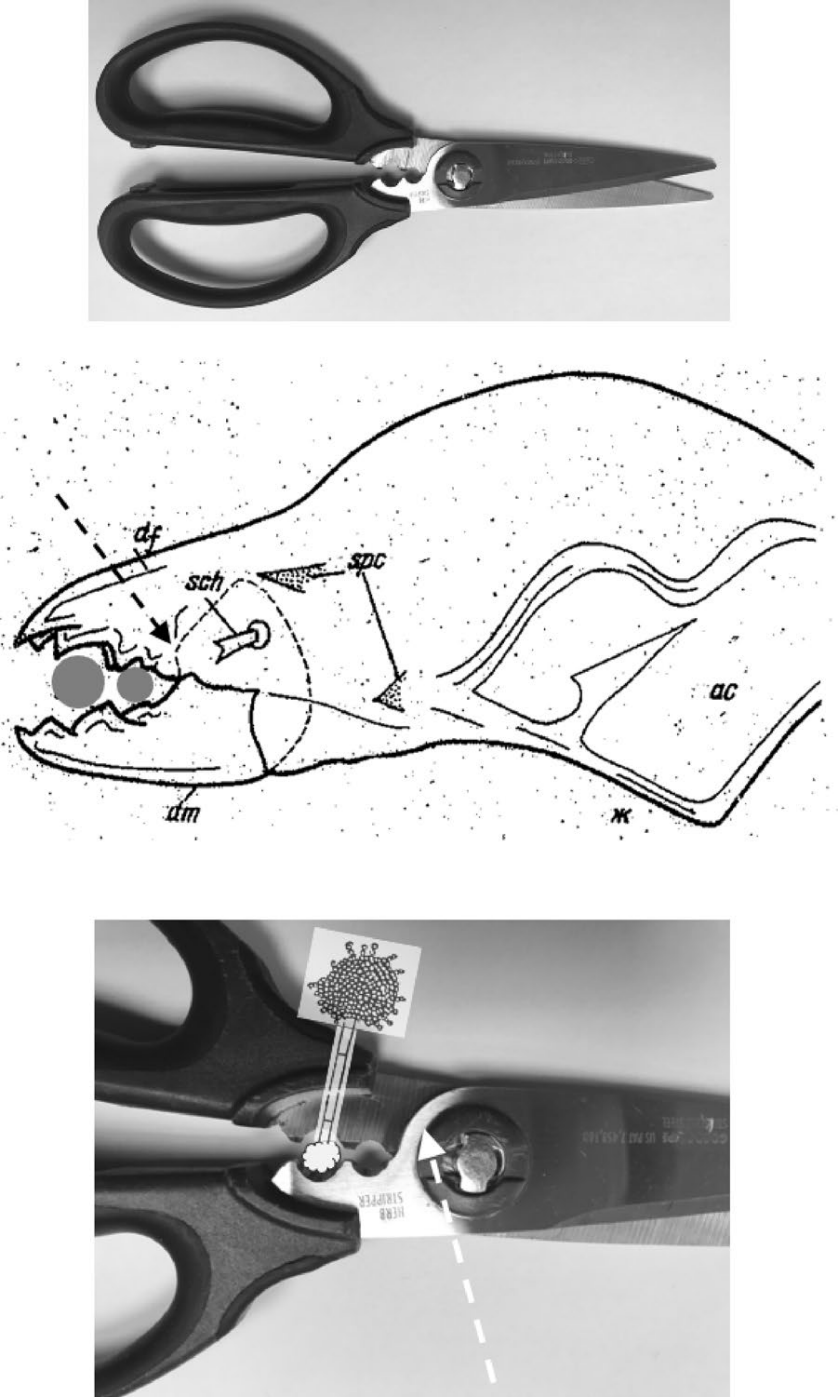
Gleaning chela of *Tyrophagus putrescentiae* may strip fungal spores from hyphal stalks. Upper, herb-stripping scissors, note reinforced notches near axil of handles. The scissor blades can cut, but the whole tool can be reversed for another use. Middle, chela (with original abbreviations) amended from Akimov (1985) with permission. Grey circles suggest hyphal stalks in cross-section within two ‘notches’. Dashed arrow = bulging ascending ramus posterior of mastication surface. Lower, likely feeding action by which the hypha will pass through a proximal socket and the fungal spores be stripped off its head during substrate browsing if the scissors were miniaturised (and the chelal tips are to the left). Overlay amended from PSM_V09_D432_Eurotium_aspergillus_glaccus.jpg from Wikimedia Commons ex Popular Science Monthly (1879) Volume 9 now ‘in the public domain’. Note heavy shiny reinforcement behind notches around blade rotation point (like an ascending ramus) indicated by white dashed arrow

Compared to an assumed null of a circular basal ramus of radius *L*1*U* centred on the condyle (i.e., $nbO[VR]=1.0$, as an attempt to deal with the jaw’s optimal inertia beyond the limits of an ideal mastication machine; Bowman (2023, 2024)), all three species whether UK wild-collected or from the laboratory show a $10\%-32\%$ increase in the observed average velocity ratio *nbO*[*VR*]. This suggest either a large morphological investment where the adductive tendon is attached, but this is small in at least *Tyrolichus casei* in Fig. 4. Or, more likely from fitting circles by-eye in Fig. 5, a differentiated ascending ramus is present. Comparing across the UK beehive species,

- This % is highest in *C. lactis* overall (*nbO*[*VR*] increase from 1.0 mean $=23\%$). This species may rely upon strengthening this particular area for its postulated fluid-skimming feeding action (Fig. 9).
- This % is lowest in *G. domesticus* (overall mean increase in *nbO*[*VR*] from 1.0 is $6\%$) commensurate with its mastication surface being like a saw (Bowman 2024).
- The $21\%$ increase from the null in *T. putrescentiae* overall suggests that posterior of the postulated nutcracker action of the central moveable digit tooth (Bowman 2024), the chela may therefore act like ‘herb-stripping scissors’ do, enabling the browsing and gleaning of material off of spike-like foodstuff (Fig. 10). As the adductive crunch force (*F*2) will be much larger here than at the moveable digit tip, some strengthening proximally would be expected.

In *T. putrescentiae*, at $\approx 7\ \mu$m for a ‘proximal notch’ size (from Bowman (2021b) and Fig. 10), this pocket would match the size of *Aspergillus* hyphae at $3-6\ \mu$m diameter (Lanzarin et al. 2015) for example. So allowing the scraping off of the columnar conidial heads (which are up to $70\ by\ 30\ \mu$m in diameter; https://www.adelaide.edu.au/mycology/fungal-descriptions-and-antifungal-susceptibility/hyphomycetes-conidial-moulds/aspergillus) and the garnering of the $2.5-3\ \mu$m fungal spores (Mousavi et al. 2016) into the gut.

Acarids eat these sorts of indoor environment fungi in laboratories, and a beehive approximates a warm inhabited domestic milieu. Consiliently, the smallest fragments observed in the astigmatan caecal lumen were estimated by Bowman (2024) as around $0.5-2\ \mu$m in diameter. Note the apparent part suppression of the central tooth in the laboratory culture T13 (Fig. 6) suggesting that feeding upon yeast and wheat germ may not need this special action.

**Fig. 11:**
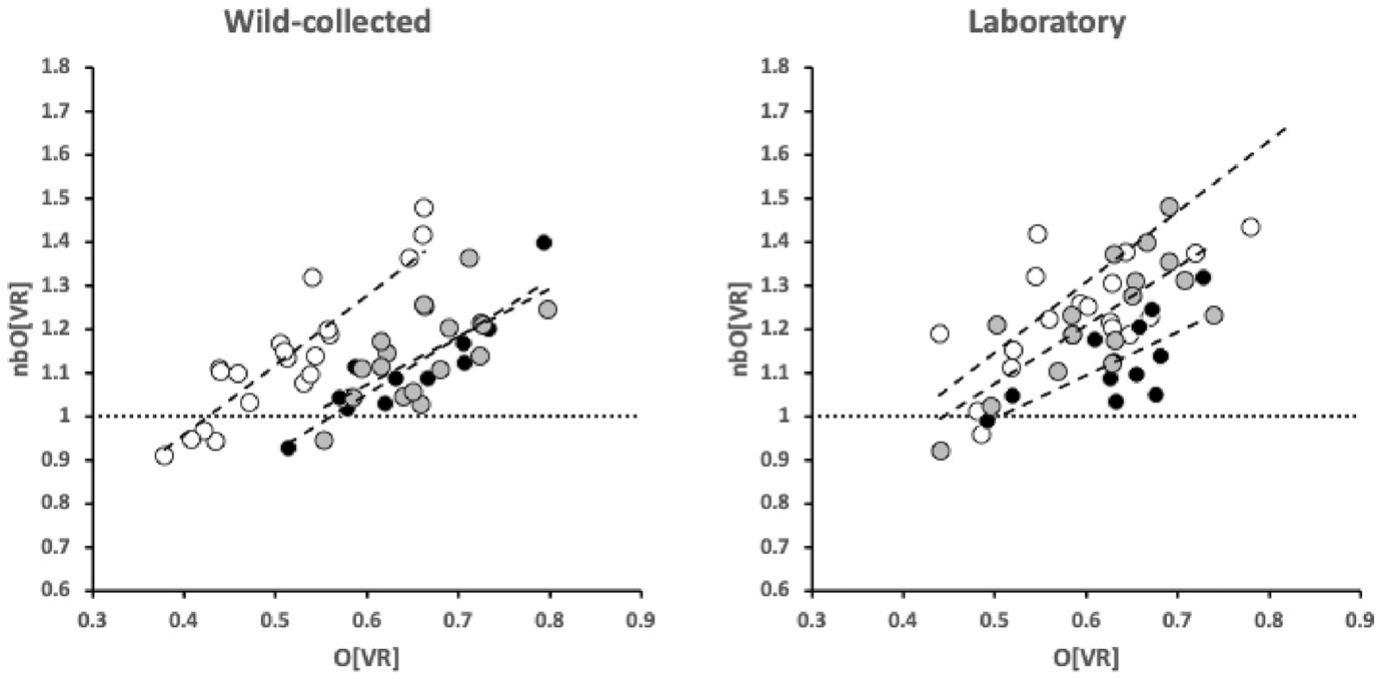
Partitioning between horizontal ramus (indicated by *O*[*VR*]) and the ascending ramus/coronoid process (indicated by *nbO*[*VR*]). Open dots = *Carpoglyphus lactis*. Grey dots = *Tyrophagus putrescentiae*. Small black dots = *Glycyphagus domesticus*. Linear regression slopes $0.9-1.6$. Note evolutionary digit shape shift in *C. lactis* due to culturing (on average change from $\frac{nbO[VR]}{O[VR]}=2.23$ wild-collected to 2.19 for the lab culture Ca4)

The relative partition of resources between the horizontal ramus (indicated by *O*[*VR*]) and the ascending+basal ramus (indicated by *nbO*[*VR*]) is broadly the same within each of the three species and their origin (Fig. 11).

The right-hand sub-figure shows how long-term culturing of *C. lactis* on yeast and wheatgerm (see Bowman 2023) shortens the moveable digit and mastication surface (reducing $VR_{tip}$) but does not dramatically affect the overall proportional shape of the digit in velocity ratio space within a cultured species.

**Fig. 12:**
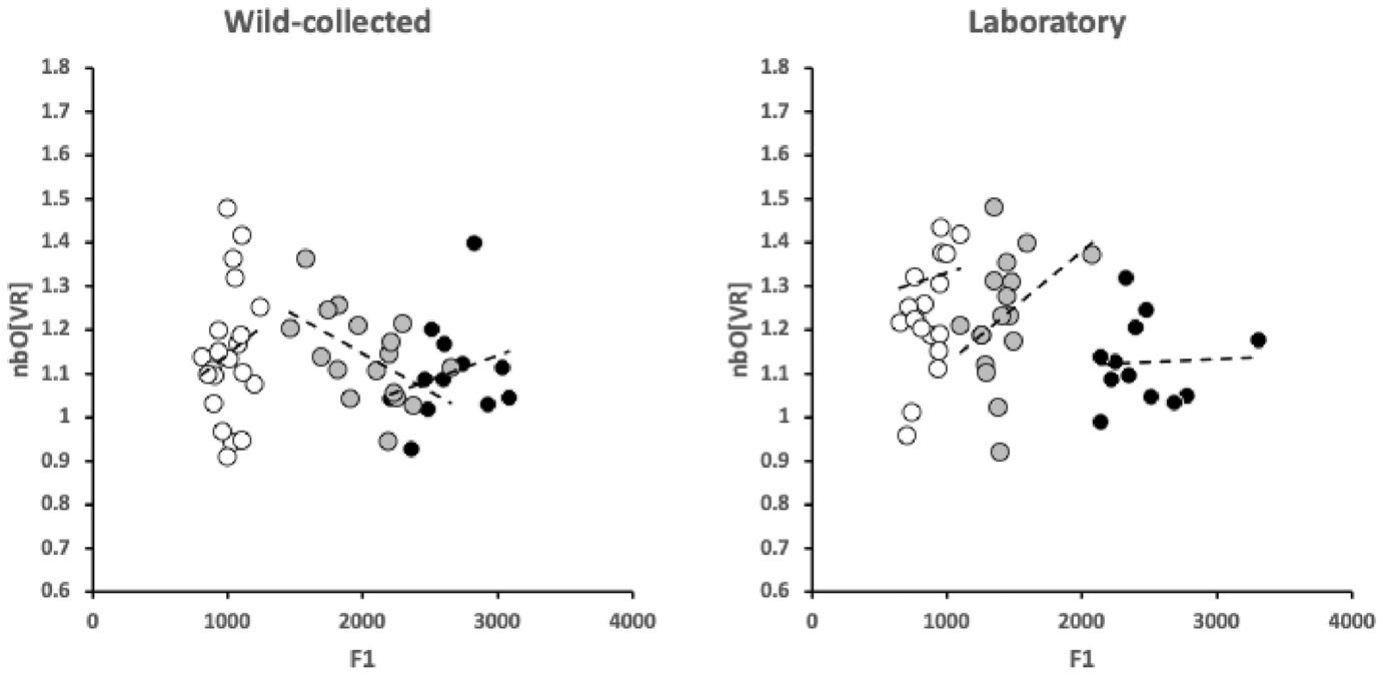
Expected lack of relationship of ascending ramus/coronoid shape in velocity ratio space (indicated by *nbO*[*VR*]) with scale of force on chelal adductive tendon (*F*1). Open dots = *Carpoglyphus lactis*. Grey dots = *Tyrophagus putrescentiae*. Small black dots = *Glycyphagus domesticus*. $R^{2}$ values left to right over the two panels $=0.0326,0.2838,0.0664;0.0017,0.1426,0.0022$

Accordingly, one infers that chelal strengthening simply matches the increased adductive forces between individuals of the same species. This is confirmed in Fig. 12 where there is no relationship between *nbO*[*VR*] and the primary adductive force on the digit’s closing tendon (*F*1; $R^{2}$ ranging from $0.0017-0.0238$) within or between taxa. This is expected within species given that the velocity ratio calculation ($VR=\frac{L1U}{L2M}$) itself incorporates any increase in *L*1*U* with any overall size-driven increased cheliceration between individuals of the same species. What matters to the velocity ratio is the elongation or shrinkage of *L*2*M* evolutionarily between species. Here the left hand subfigure of Fig. 11 shows that the relative shape of wild-collected moveable digits in *C. lactis* is different from that common relative form of the acarid and glycyphagid. This evidences the suggestion of a markedly different style of feeding in the wild for the carpoglyphid.

### Overall Hypothesis test - Is *Tyrophagus putrescentiae* an intermediate trophic form?

**Table 3:** Proximal (upper panel) and Distal (lower panel) astigmatan digit surface results. Ratios listed under ‘Interpolate @’ are for that *Value* distance along the geodesic from consensus $\varOmega$ for *G. domesticus*, given as weights [$\varOmega$ for *Carpoglyphus lactis* : $\varOmega$ for *Glycyphagus domesticus*] in Fig. 3. Equivalent distance along this geodesic from consensus $\varOmega$ for *C. lactis* would be relevant $lengeodesic-Value$. Note, $1.645\sigma _{proximal}=6.474142$ and $1.645\sigma _{distal}=6.091804$ indicating the acceptable ‘thickness of the regression line’

Variable	Value	Meaning		
$\sigma _{proximal}$	3.935649	typical error		
$lengeodesic_{proximal}$	10.53553			
*lencheck*4	6.919204	$\rightarrow$ *T. putrescentiae*	Wild-collected	(*)
Interpolate @	3.00262694	to *G. domesticus*	[0.57:1.43]	
*lencheck*4	6.331303	$\rightarrow$ *T. putrescentiae*	T13 laboratory	(ns)
Interpolate @	3.31869294	to *G. domesticus*	[0.63:1.37]	
$\sigma _{distal}$	3.703224	typical error		
$lengeodesic_{distal}$	10.06849			
*lencheck*4	6.763756	$\rightarrow$ *T. putrescentiae*	Wild-collected	(*)
Interpolate @	2.41643876	to *G. domesticus*	[0.48:1.52]	
*lencheck*4	6.329194	$\rightarrow$ *T. putrescentiae*	T13 laboratory	(*)
Interpolate @	3.27226082	to *G. domesticus*	[0.65:1.35]	

Table 3 shows that both proximally and distally the wild-collected *T. putrescentiae* sample in its chelal moveable digit form is not a simple intermediate between the consensus form of the other two species. Only part of the moveable digit *pattern* of the laboratory sample of *T. putrescentiae* could be considered as ‘sitting on the regression line’. One concludes that on the comparative axis in Fig. 25(c) from Bowman (2021b) or indeed herein in Fig. 2, the moveable digit form of *T. putrescentiae* in the wild is distinct from any inter-taxon smooth gradation. Astigmatans do vary further in the detailed *pattern* of trophic adaptations over their ground space of cheliceral and chelal adaptations described by Bowman (2021b).

## Discussion

This, as a functional ecomorphological (Feilich and López-Fernández 2019) study, *per force* examines what Nature sees in the phenotype determining survival i.e., ‘fitness‘ here determined by the efficiency of the design of a mite’s food-grasping tools. How things actually work in practice is crucial in posing any evolutionary argument. This discussion will start simple and hopefully successfully take the reader on a journey whereby levels of complexity are slowly built up and up in order to aid intuitive understanding, rather than leaping straight to the conclusions from at first sight seemingly impenetrable mathematics.

### Pre-amble

The summary observed average velocity ratio over the mastication surface *O*[*VR*] is offered to encompass how jaws might work comparatively. Regarding the evolution of digit form, the method herein explores shape change when the moveable digit is scaled to the same output lever arm sizes i.e., *L*2*M* is partialed out. So it leaves aspects of any relative change in input lever moment arm (and comparative input forces) intact.

**Fig. 13:**
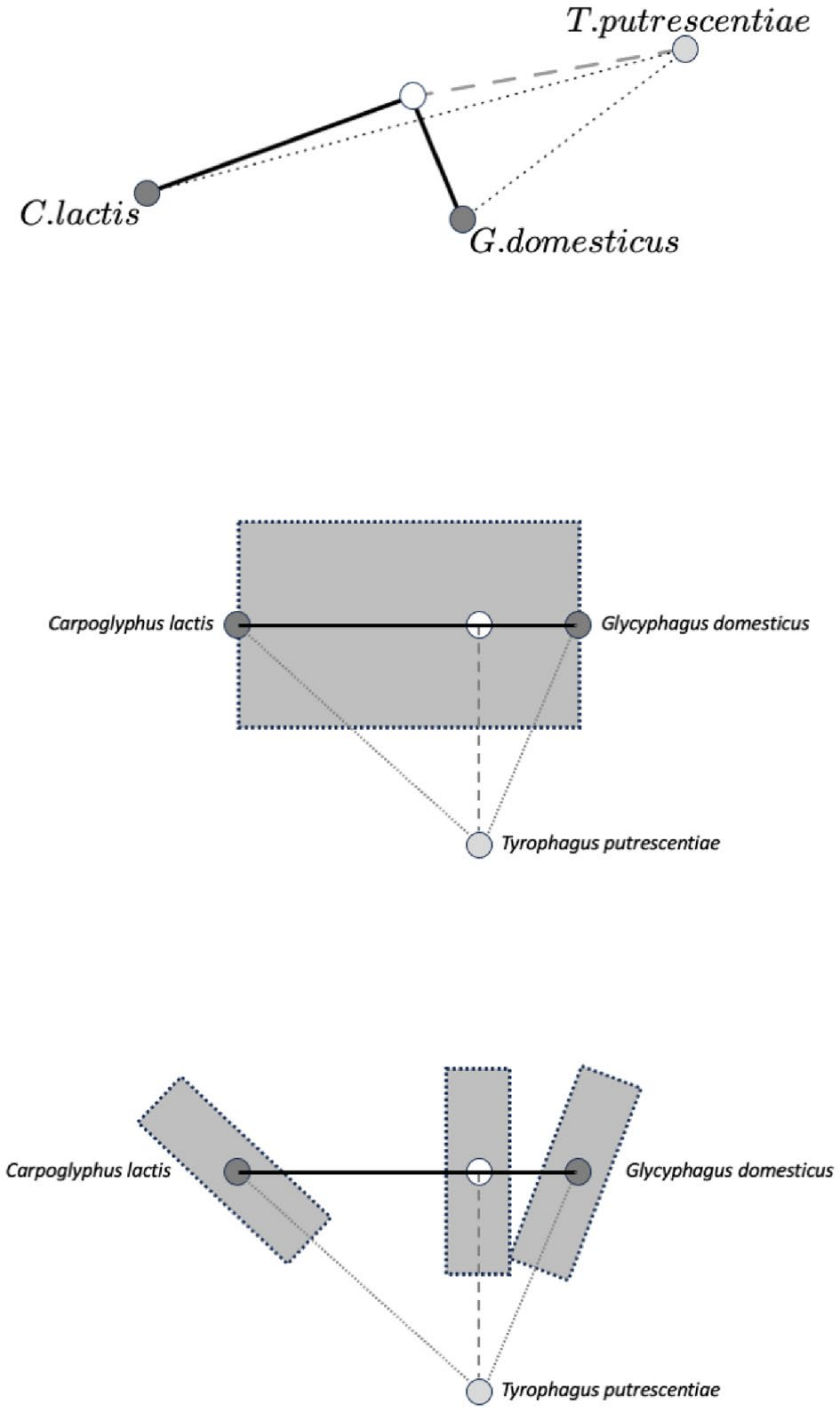
Three views illustrating typical moveable digit profile results (excluding the proximal data set for laboratory sample T13) as comparative distances. Dark grey dots = consensus $\varOmega$ matrices for each reference taxon. Open dot = nearest matrix interpolate to a specified third $\varOmega$ ‘test’ matrix for *Tyrophagus putrescentiae* (pale grey dot). The curved geodesic between the consensus reference species shown as a heavy line (size = *lengeodesic*). Dashed grey line indicates estimated minimum distance geodesic (*lencheck*4) to the ‘test’ species. Dotted lines illustrate the remaining inter-taxa distances. Upper, 3D non-planar arrangement of taxa (’broken-stick’ geodesic for exemplification purposes only). Middle, ‘rolling’ projection along the geodesic from above showing uniform ‘fatness’ of critical zone around curved geodesic. Lower, planar projection from above showing how the critical zone appears to bend (and possibly appears to shorten) as the curved geodesic progresses. The $\varOmega$ matrix for each consensus reference taxa accordingly are always significantly different than the ‘test’ matrix if the interpolated matrix is

Here the overall ‘thickness of the matrix admixture regression lines’ ($\approx 6.2$ units) is a large fraction of the general scale of the length of the geodesics (aka *lengeodesic*) at $\approx 10.3$. Also, the *pattern* of *Tyrophagus putrescentiae* is near (at $\approx 6.6$ overall) a ‘matrix interpolate’ which is closer to that of *Glycyphagus domesticus* (at $\approx 3.0$ units overall along the geodesics) than that of *Carpoglyphus lactis* (at $\approx 7.3$ overall along the geodesics). Figure 13 scaled to the overall size of results shows this as a summary.

The location of the reference taxa are indeed fairly close to each other ($\frac{lengeodesic}{1.645\sigma }\approx 1.5$) on the manifold (yet they would be still considered as statistically distinct). While laboratory culture T13 may not be significantly displaced from its nearest interpolate on the geodesic, it can still have a *pattern* that remains different than that ‘just’ for *C. lactis* or that ‘just’ for *G. domesticus*. Arranging taxa in an ordination simply based upon their inter-taxa distances (as say points on a metric multidimensional scaling 2D ordination often used by morphologists) does not necessarily unlock all of the information regarding their evolution. Putative transitional paths matter.

**Table 4:** Interpoint Riemannian distances for $\varOmega$ matrices of UK astigmatan beehive species. Note the within consensus variation is represented by $\sigma$ in Table 3. T13 is the laboratory culture of *Tyrophagus putrescentiae*

	*C. lactis*	Lab	T13	Wild	Wild	*G. domesticus*
	Consensus	Interpolate		*T. putrescentiae*	Interpolate	Consensus
PROXIMAL						
*C. lactis*						
Consensus	0	7.22	9.42	2.81	7.53	10.54
Lab						
Interpolate	7.22	0	6.33	6.93	0.32	3.32
T13	9.42	6.33	0	7.04	6.41	8.02
Wild						
*T. putrescentiae*	2.81	6.93	7.04	0	6.92	7.73
Wild						
Interpolate	7.53	0.32	6.41	6.92	0	3.00
*G. domesticus*						
Consensus	10.54	3.32	8.02	7.73	3.00	0
DISTAL						
*C. lactis*						
Consensus	0	6.80	9.87	2.80	7.65	10.07
Lab						
Interpolate	6.80	0	6.33	6.83	0.86	3.27
T13	9.87	6.33	0	6.98	6.41	7.33
Wild						
*T. putrescentiae*	2.80	6.83	6.98	0	6.76	7.27
Wild						
Interpolate	7.65	0.86	6.41	6.76	0	2.42
*G. domesticus*						
Consensus	10.07	3.27	7.33	7.27	2.42	0

Inspection of Table 4 shows that irrespective of examining the moveable digit features proximally or distally

- the two reference consensus matrices are far apart as expected
- the two interpolated $\varOmega$ matrices are indeed close to each other overall on their common defined transitional geodesic suggesting that the analytical process used herein does indeed map to a generally common intermediate for the acarid species general form even though the ‘test’ matrices are clearly different (i.e., the ‘knot’ has worked)
- the interpolated matrices for the laboratory sample and that for the wild-collected sample are around the same distance along their individual geodesics (i.e., the length of *lengeodesic* is similar for each digit region). The species distinction ‘signal’ is coming from both regions consistently.

Yet the distance from each ’test’ matrix to the consensus matrices of the pair of reference species swops over. T13 is slightly closer to $\varOmega _{Gdomes}$ than to $\varOmega _{Clactis}$ while the wild-collected *T. putrescentiae* is close to $\varOmega _{Clactis}$. This all can only be true if the direction of the vectors from the interpolates’ position to each test matrix effectively point in different directions, like someone using flag-based semaphore signalling (https://en.wikipedia.org/wiki/Flag_semaphore) in 3D.

**Fig. 14:**
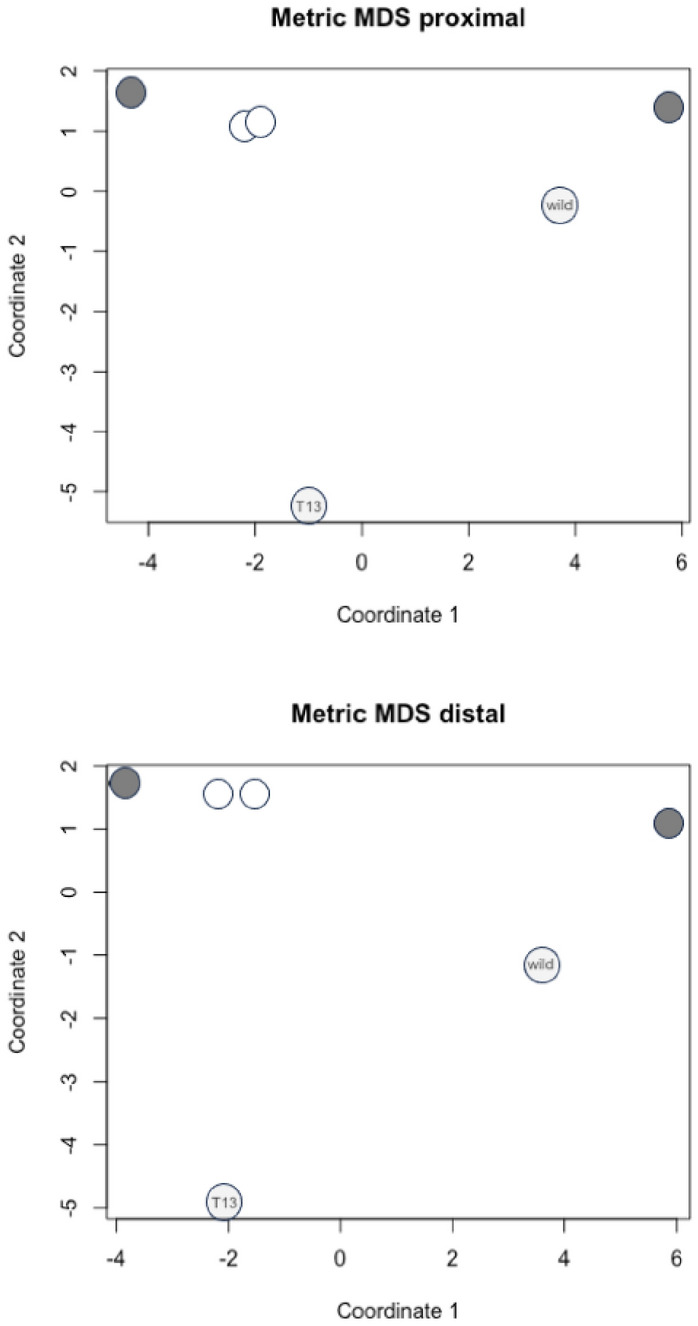
Classic 2D multidimensional scaling of Riemannian distances in Table 4 (using cmdscale in R) for moveable digit projective locations proximal and distal to the condyle. Dark grey dots = consensus reference taxa, *Glycyphagus domesticus* top left, *Carpoglyphus lactis* top right. Open circles = transitional matrix interpolates, that for wild-collected acarids is slightly to the left, that for T13 is slightly to the right. In reality the $\varOmega$ for the wild-collected *Tyrophagus putrescentiae* moveable digit (pale grey dot) is significantly different from its transitional interpolate despite its apparent proximity to it, while T13 (pale grey dot basally) despite its apparent isolation is not significantly different to its transitional intermediate. In other words the location of T13 is actually proud above the 2D plane of this page, and the wild-collected $\varOmega$ is located deep within the page (or vice versa), with the page being tilted somewhat around the ‘thickened’ path from one reference taxon to the other

Indeed, apparently optimally projecting onto a flat plane (Fig. 14) can be misleading since in this figure the laboratory sample T13 looks as if it has a significantly different $\varOmega$ to the implied geodesic between the reference taxa, and the wild-collected *T. putrescentiae* sample looks like it has a non-significant $\varOmega$. This error would be concluded too by using ‘Euclidean common-sense’ logic (see Introduction) on the values in Table 4. Both of these false conclusions are not evidenced on a properly curved surface - acarologists beware!

### What do the fitted intermediate chelal profiles look like?

**Fig. 15:**
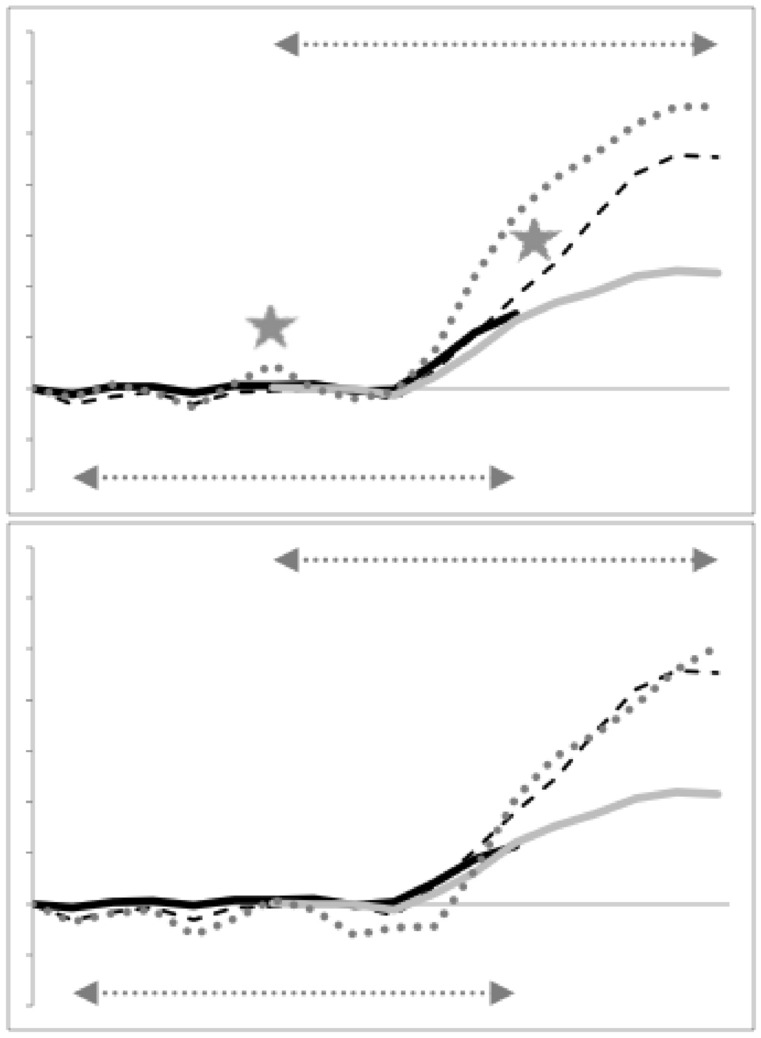
Estimated moveable digit profiles (see Fig. 4) at closest point to *Tyrophagus putrescentiae* along the reference geodesic. Dotted line = observed profile. Dashed line = simple average over the individuals from two reference taxa. Black solid line = reconstructed profile for distal segment of moveable digit at nearest interpolation point on the consensus reference geodesic. Grey solid line = reconstructed profile for proximal segment of moveable digit at nearest interpolation point on geodesic. Upper, wild-collected acarids, with points of difference marked by grey stars. Lower, T13 showing less distinct central tooth

Getting a feel for the profiles is an important early step. Labelling the matrices interpolated between the two reference taxa for each test *T. putrescentiae* matrix as $\varOmega _{int}$ in turn, then one can estimate a typical chelal profile ($\widehat{y_{int}}$) at these points by searching over the digit’s *p* multidimensional space (both negative and positive values) to minimise a simple root mean square norm like $||\varOmega _{int}-\widehat{y_{int}}.\widehat{y_{int}}^T||$. Herein the sum of the squared differences over all $p\ by\ p$ entries was used and the observed (1 row by *p* column) mean profile over reference individuals for that chelal region was used as an appropriate unbiased start position. This yields Fig. 15. If the objective function was divided by $p^2$ and square rooted it would be a *rms* (root mean square) Euclidean distance.

One can see

- a very good agreement in the estimated profiles for each moveable digit region through the ascending ramus ‘knot’
- that the estimated profile for the horizontal and ascending ramus as expected matches well the simple average over the reference individuals’ data
- that the relative posterior positioning of the ascending ramus in *C. lactis* (grey star in Fig. 7 left panel) engenders a flatter interpolated digit profile between the two consensus taxa configurations over the basal ramus region
- that the scale of the ‘significance/non-significance’ of the differences between the acarid profiles and the smooth transition between the carpoglyphid and glycyphagid consensus forms is mirrored by the relative elevations of the observed data shape versus the interpolated profile (indicated at the grey stars).

It is a trivial assertion to state that it is impossible for the profile of a ‘protruding’ central tooth in *T. putrescentiae* to be between the profiles of a lower asperity height *G. domesticus* profile and that of an even smoother bar-like profile for *C. lactis* (see Figs. 6, 7 and 15). This is the origin of the significance of the results for the distal region in Table 3. This region must have evolved uniquely for the acarid despite the common [‘dip’, ‘up’...] patterning of these astigmatan moveable digits.

However, the acarine chela is all one morphological system as a whole whose design is driven by all of the physics involved. Although evolution does not necessarily take the optimal route from form to form Rosen (1967), Fig. 16 shows how the estimated geodesic path does smoothly deform the proximal regions of the digit between the species in a plausible scenario as to how this particular region might have changed under trophic selection pressures.

**Fig. 16:**
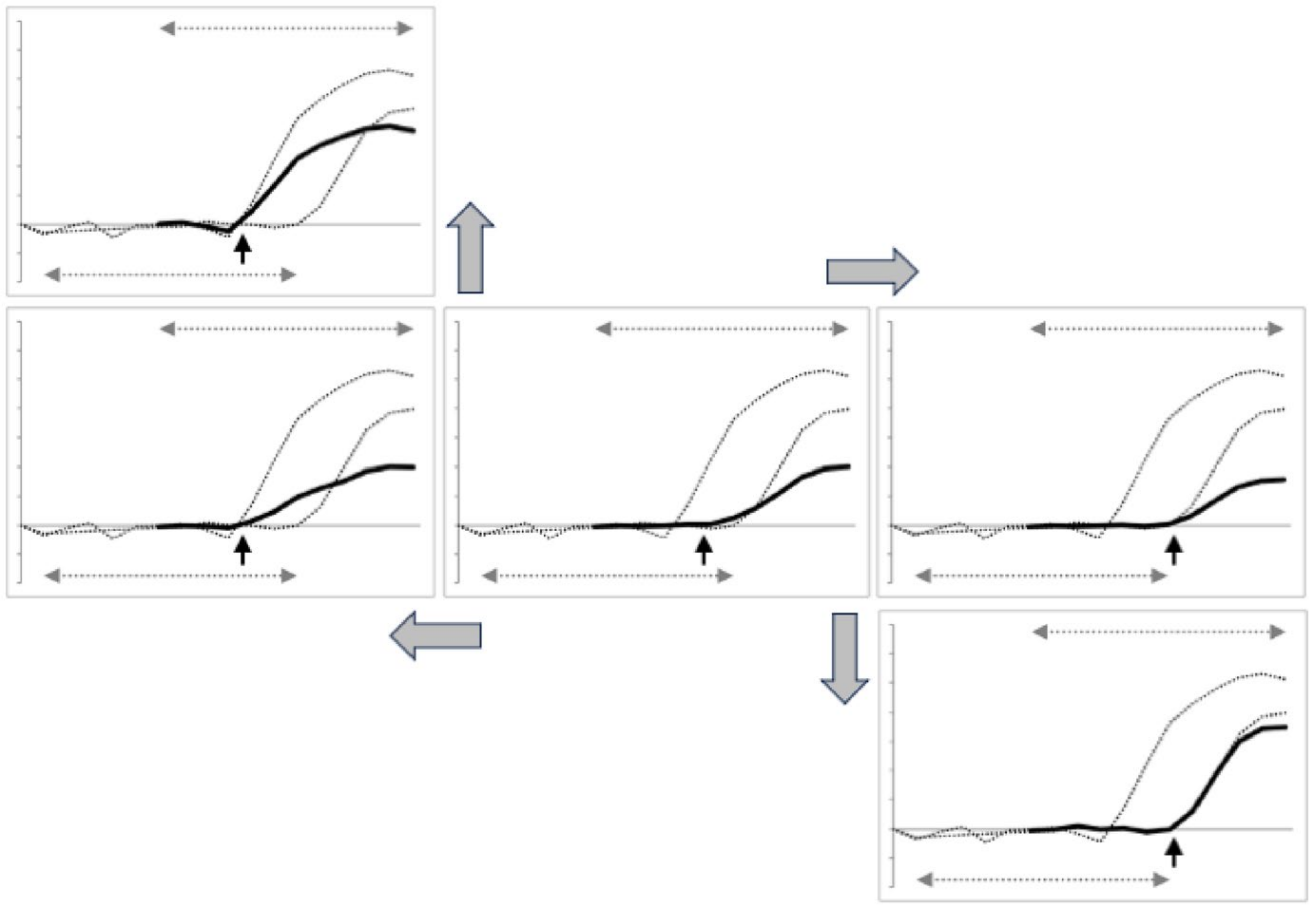
Plausible evolutionary path for proximal region of moveable digit (see Fig. 4) from a plesiomorphic bar-like form (centrally represented by an optimal reconstruction from an admixture combination of $1.25\ \varOmega _{Clactis}: 0.75\ \varOmega _{Gdomes}$). Large grey arrows = indicative transitions. Small black arrows = inflection points for ascending ramus commencement. Dotted profiles are *Glycyphagus domesticus* average and *Carpoglyphus lactis* average over individuals as final ‘targets’. The reconstructed profile from an extreme top left admixture $=1.975\ \varOmega _{Clactis}: 0.025\ \varOmega _{Gdomes}$ (not shown) approximates well that of consensus *G. domesticus*

That is, starting from a plesiomorphic bar-like digit plus a minor coronoid process centrally in Fig. 16 (as suggested for other animals DeMar and Barghusen 1972), one can see

- first, relative shifts of the profile inflection points (i.e., where the ascending ramus starts) left or right, and
- second, the deformation to a rounder basal ramus (for *G. domesticus*, top left) and a deformation to a more abrupt sigmoid shaped ascending ramus for *C. lactis* (bottom right).

This supports the horizontal ramus shortening in shape (middle row left, matrix admixture $= 0.75\ \varOmega _{Clactis}: 1.25\ \varOmega _{Gdomes}$) followed by the coronoid process being optimised (through its growth, top left, matrix admixture $=0.25\ \varOmega _{Clactis}: 1.75\ \varOmega _{Gdomes}$) for the evolution of *G. domesticus*, while the horizontal ramus lengthens in shape (middle row right, matrix admixture $=1.75\ \varOmega _{Clactis}: 0.25\ \varOmega _{Gdomes}$) followed by again some (different) coronoid growth (bottom right, matrix admixture $=1.995\ \varOmega _{Clactis}: 0.005\ \varOmega _{Gdomes}$) for the evolution of *C. lactis*. It would seem that the velocity ratio of the tip ($VR_{tip}$) is probably selected for different feeding advantage (see Bowman 2021b) first from any putative ambulacral seta, before the inertial mechanics of the digit is optimised with ascending ramus modifications (and a full chela formed with appropriate accompanying fixed digit development).

**Fig. 17:**
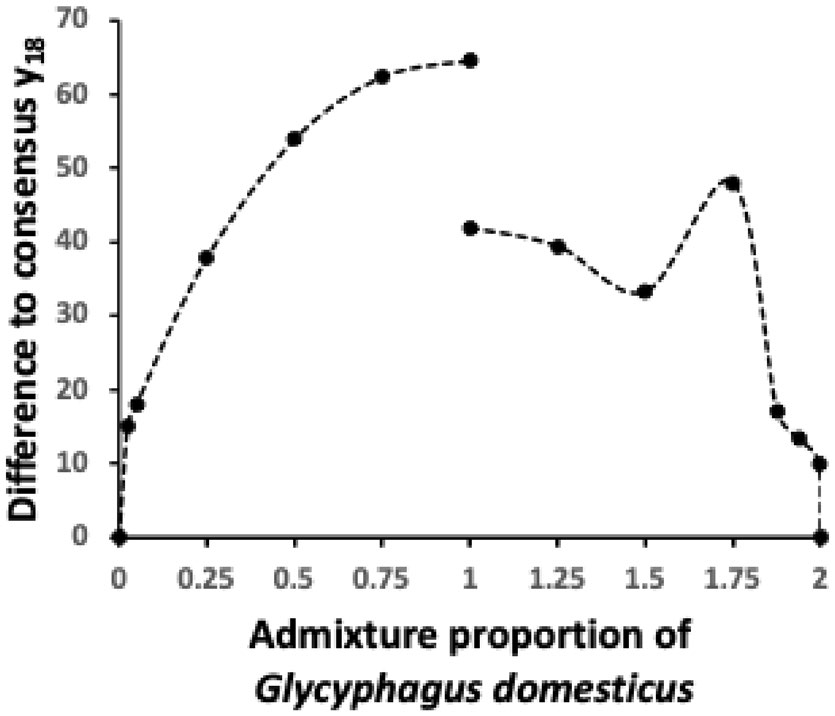
Difference at projective location $y_{18}$ semi-landmark between the proximal region reconstructed profile at the interpolated $\varOmega$ matrix on the geodesic between the two reference taxa $\varOmega$ matrices at specific admixture proportions. Dashed line to the left as one approaches the *Glycyphagus domesticus* matrix - note overall steep curve. Dashed line to the right as one approaches the *Carpoglyphus lactis* matrix - note overall shallower curve. Anomaly on the right trajectory is a computational artefact due to the swopping of the reconstruction from an essentially positive bar-like shape to an essentially negative bar-like profile (as one ‘loses’ the signal of any teeth)

In this way, this in astigmatan mites is an example of a “Local Evolve - Global Rescale” model (Goldin et al. 2008) where a rapid change in gape (and therefore possibly also in reach) is the primary evolutionary novelty locally in the basic profile, around which the mechanical advantage of the system is more slowly deformed to preserve the digit general shape and the global constraints are re-optimised. The latter will be visually exaggerated by overall cheliceral or body size increases as $mass \propto length^{3}$. Indeed the radius of the basal ramus since the angle $\alpha$ illustrated in Fig. 11 of Bowman (2024) can vary (if it is assumed to be symmetrical around the condyle) $= min(L1U, max(y))$ where *y* is in actual not rescaled units i.e. in $\mu$m. From previous results (Bowman 2021b) this should broadly scale with body size. Indeed the differences of admixture weights to well approximate the matrices near each consensus reference point shows that the relative steepness (and thus perhaps the selection pressure) of the manifold along the geodesic is different at each end. This is illustrated in Fig. 17.

Recall that during any evolutionary transition mites still have to effectively feed, there cannot be non-functioning “Hopeful Monsters”!

### Does moveable digit profile bending energy agree?

Recall that visually the shape of the astigmatan ascending ramus (i.e., posterior of the mastication surface on the horizontal ramus) up and over the basal ramus to form the ‘coronoid process’ is different in the three beehive collected species (Fig. 5). The glycyphagid for instance is more rounded.

Now, bending energy has been used in shape characterisation for many years (e.g., Cesar and Costa (1997)). Considering it is the next step in complexity. The bending energy of an object denotes the energy stored in its shape (i.e., its potential energy on subsequent unfolding). The Explanatory appendix outlines the derivation of the (moveable digit) profile bending energy.

**Fig. 18:**
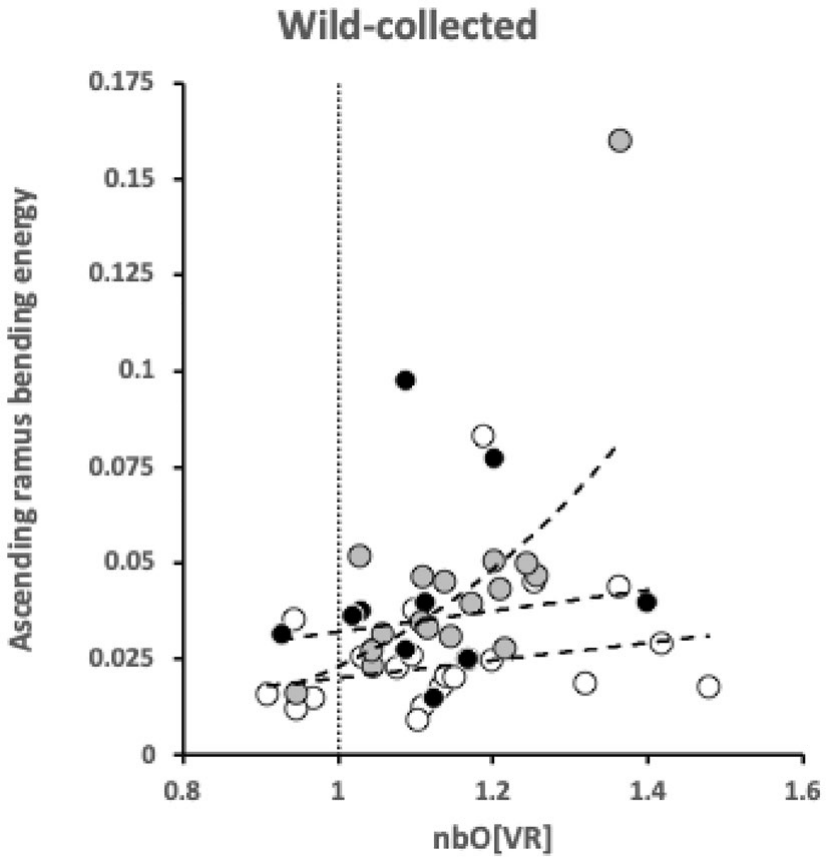
Divergence from theoretical circular coronoid process (of radius *L*1*U*, dotted boundary) correlates with higher bending energy of moveable digit profiles. *nbO*[*VR*] = Observed average velocity ratio of moveable digit profile posterior of mastication surface. Vertical dotted line = null equivalence. Open dots = *Carpoglyphus lactis*. Grey dots = *Tyrophagus putrescentiae*. Small black dots = *Glycyphagus domesticus*. Indicative power regression lines

Fig. 18 shows that the more asymmetric (away from a circle’s segment shape) an ascending ramus is (i.e., $nbO[VR]>1$), the greater the increase in moveable digit (profile) bending energy.

*T. putrescentiae* can have a particularly unusual shape (c.f. Fig. 5) suggesting extra evolutionary differentiation may be occurring in this region (see Fig. 10).

Note such sampled 2D curve bending energy is not magnification invariant (Bowie and Young 1977), actual size matters. One possibility for its normalisation is that given $BE(circle)\propto circle\ area$ then a possible scale invariant comparative measure (following Cesar and Costa 1997) for the ‘shape complexity’ of the horizontal rami between individuals would be

$$\begin{aligned} SC_{hr}=\frac{{\widehat{BE}}_{tip-x_{i_{e}}}}{m^{2}} \end{aligned}$$

(where *m* = *length of mastication surface or ‘drape distance’’*, Bowman (2024) and $x_{i_{e}}$ is the semi-landmark at the posterior end of the mastication surface). A similar normalisation to a circle of the same perimeter as the profile length could be done for the ascending ramus up and over the coronoid process if the ‘chain distance’ ($m_{ar}$) from $x_{i_{e}}$ to $x_{18}$ was estimated. Table 1 gives these $m_{ar}$ figures.

The mastication surface shape complexity is highest ($\mu =148.6, SD=85.49$) in *T. putrescentiae* as expected given its heterodonty. It is lowest ($\mu =16.3, SD=10.23$) in *C. lactis* as expected given its almost plesiomorphic form. Of course, such edentulism may have complicated evolutionary origins (Yang and Sander 2018). *G. domesticus* showed a mean mastication surface shape complexity value of 65.3 ($SD=3.06$) confirming its greater similarity with the acarid (as previously found by Bowman (2024)). Note that there is only mild evidence that body miniaturisation (as indicated by diminished idiosomal index *IL*) may be driving the progressive loss of noticeable digit teeth between the wild-collected species ($R^{2}=0.2595$). Such has been implicated in bird beak evolution (Wang et al. 2020).

The shape complexity of the ascending ramus up and over the coronoid process is much more similar across the three species. It is highest ($\mu =98.1, SD=135.21$) in *T. putrescentiae* and lowest ($\mu =58.9, SD=39.94$) in *G. domesticus* with a mean value of 90.2 ($SD=65.47$) for *C. lactis* again confirming the comparatively ‘anomalous’ distinct proximal shape of the acarid (see Fig. 6).

Calculating $\frac{SC_{ar}}{SC_{hr}}$ and averaging shows that this ratio is highest ($\mu =6.6, SD=4.48$) in *C. lactis* confirming this species’ major differentiation compared to the low (and similar) values for the moveable digit in the other two species ($\mu =0.6, SD=0.39$ for *T. putrescentiae*, $\mu =1.0, SD=0.59$ for *G. domesticus*).

Scaled bending energy of the digit profile is of use to acarologists. Note that so far the second moment of area (${\mathbb {I}}$) for the horizontal ramus and separately for the ascending ramus was assumed constant across species (i.e., in some sense the differential thickness of the main digit body does not yet matter, but see below for changing this simplification).

### Can considering the bending moment of a cantilever beam be useful to understand moveable digit form?

**Fig. 19:**
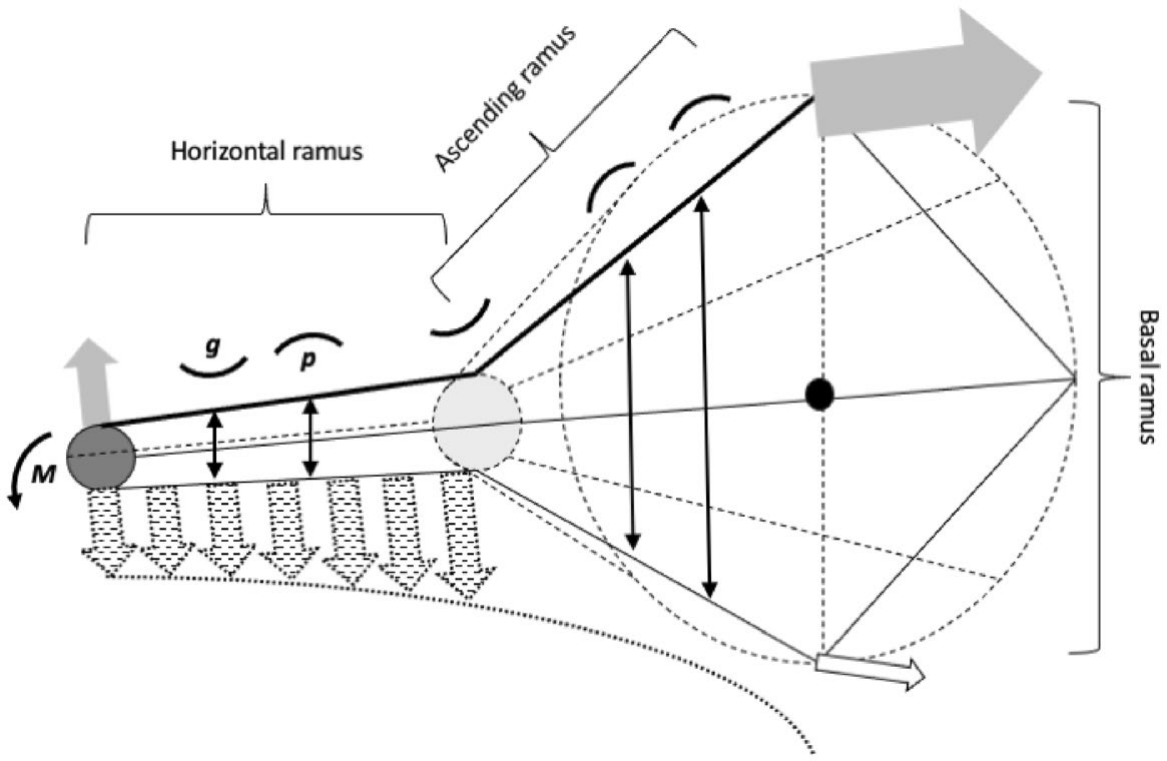
Stylised moveable digit (see Fig. 4) as a cantilevered composite width-varying rod-like beam fixed at the condyle (black circle). Dark grey circle = moveable digit tip. Pale grey circle = junction of horizontal ramus and ascending ramus at end of mastication surface (i.e., at $x_{i_{e}}$ along *L*2*M* axis). Double headed arrows are diameter of the rod at points where curvature (solid black curved segments) is illustrated above the dorsal edge of moveable digit (heavy black line, straight here just for illustration). g = gullet. p = peak. Grey arrows are adductive tendon *F*1 force at distance *L*1*U* from the condyle with the induced force *F*2 at the digit tip (and thus its equivalent static load when biting food on chelal occlusion). Hashed dotted arrows = set of induced loads (with hyperbolic envelope) yielding overall bending moment (*M*). Note no induced reactive force from foodstuff proximal of posterior end of mastication surface. Open arrow is abductive tendon force at similar distance (*L*1*L*) from the condyle

The previous section is justified by the theory of one dimensional curves, this section that follows herein is the next step of complexity - allowing measures to have depth i.e., to become two dimensional. This relies upon the continuum solid mechanics of the fabric of the moveable digit (Fig. 19).

This is derived in detail in the Explanatory appendix. Estimating Young’s modulus (E) allows one to consider the strength of objects.

Using this approach, by making an assumption of fixed bending moment at the condyle (*M*) for a species as an algebraic constraint (whereby changes in chelal digit flexural rigidity are compensated by matching changes in curvature for individual specimens), Table 1 shows that the ascending ramus of all three species has a many times higher estimated Young’s modulus than the horizontal ramus. That is, it is a relatively strengthened material. Note between species comparisons of each measure should not be made (as one is simply partitioning up a species level assumption about its fixed bending moment *M*). However, the ratio between the two regions (ascending ramus to horizontal ramus) given the different *M* values for each species can be compared. That suggests that the relative ability of the ascending ramus (compared with its matching horizontal ramus) to resist stretching or deformation is notably high on average in *T. putrescentiae* commensurate with the proposed reinforced ‘herb-stripper’ function in that part of its chela (Fig. 10). A higher ${\hat{E}}$ ratio value is also seen on average for *C. lactis* compared to *G. domesticus* again consilient with local enforcement for ‘ram-slicing’ when skimming in the former (Fig. 11) and the lack of the need for reinforcement here in a saw-blade for the latter species.

Knowing the relative scale of Young’s modulus for mite structures is useful for comparative morphologists.

### Where might the horizontal ramus need more strengthening?

The previous section points to likely strength differences amongst the moveable digits. However, one seeks a mathematical equivalence class for the explanation of digit ramus shape (particularly the ascending ramus) in mites which is based on the notion of bending energy (i.e., equal amounts of stored energy) per shape depth which does not rely upon simple assumptions regarding *M* (as in the previous section). If one had this then morphological comparisons would be made more fairly.

How to do this? Any animal that tears at its food needs to have the equivalent of strong neck muscles where the head meets the body as in crocodiles, pliosaurs etc., and a jaw that can resist breaking. Mites are not different. A follow-up study as to how glycyphagids (and pyroglyphid durophages) might strengthen their gnathosoma posteriorly so as to be able to twist it (itself or indirectly by idiosomal torque) after gripping food stuff in their chelae is needed. More detailed observations by acarologists of live feeding in individual mites would also help. However, physics may help understand where mite digits themselves need strengthening against breakage or buckling when masticating.

Now, at chelal occlusion when biting on food, one could consider the moveable digit like a cantilever beam (i.e, fixed to the chelicera at the condyle, and free at its tip). Then the static force *F*2 upwards at the moveable digit tip from the adductive musculature is matched by an equal and opposite opposing force from the food (Newton’s Third Law). In that instant, this induces a static load (${\mathfrak {L}}$) on the tip of the stationary beam downwards (i.e., transversely to its longitudinal axis). This load is the force times the distance to that fixed point i.e., *F*2.*L*2*M*. However $F2=F1.VR$ (where the velocity ratio $VR=\frac{L1U}{L2M}$ and *F*1 is the input lever moment arm adductive force). So the load on the tip of the beam furthest from the fixed condylar point ${\mathfrak {L}}=F1.L1U$. Such a load induces an internal deforming moment (strain) arising from the stress.

*Just for the "ardent numerical modellers"*, one can be more explicit as follows. In a thin beam, the curvature at a point is proportional to the bending moment (Palmer and Pámpano 2020), i.e., $M=\kappa .E.{\mathbb {I}}$ (see Explanatory appendix). So, now imagine a downward force $F2_{(i=1)}$ applied at the tip of a cantilevered (fixed at the condyle) cylindrical beam of length *L*2*M*. The force applies along the whole length and the (*induced*) bending moment linearly rises from zero at the tip to a maximum at the condyle. Following https://home.engineering.iastate.edu/~shermanp/STAT447/STAT%20Articles/Beam_Deflection_Formulae.pdf, the resultant deflection $\delta (X)$ at each location $X=0...L2M$ *moving forwards* away from the condyle is

$$\begin{aligned} =-\frac{F2_{(i=1)}.X^{2}}{6.E.{\mathbb {I}}[X]}.((3.L2M)-X)) \end{aligned}$$

which at the tip (i.e., $X=L2M$) is now at a maximum $|\frac{F2_{(i=1)}.L2M^{3}}{3E.{\mathbb {I}}[X(=L2M)]}|$ downwards. Recall that the fixed load $F2_{(i=1)}$ i.e., for the moveable digit tip is $F1.\frac{L1U}{L2M}$

Now imagine instead just a downward force $F2_{(i=2)}$ applied ${\mathfrak {T}}$ short of the tip of the cantilevered (fixed at the condyle) cylindrical beam of length *L*2*M*. Then the resultant deflection $\delta (X)$ at each location $X=0...(L2M-{\mathfrak {T}})$ arising from it *moving forwards* away from the condyle is

$$\begin{aligned} =-\frac{F2_{(i=2)}.X^{2}}{6.E.{\mathbb {I}}[X]}.((3.(L2M-{\mathfrak {T}}))-X)) \end{aligned}$$

which for $X=(L2M-{\mathfrak {T}})$ is now at $|\frac{F2_{(i=2)}.(L2M-{\mathfrak {T}})^{3}}{3E.{\mathbb {I}}[X(=L2M-{\mathfrak {T}})]}|$ bending downwards. Note that there is *prima facie* no *extra* bending contribution at $X=L2M$ any more, the beam just extends linearly distally when past the (new) load point, which itself has moved proximally by ${\mathfrak {T}}$. Note also that (ignoring the dentition as a first approximation), the fixed load $F2_{(i=2)}$ is now the increased $F1.\frac{L1U}{(L2M-{\mathfrak {T}})}$ value (i.e., perpendicular to *L*2*M*) accordingly by standard leverage rules (Bowman 2024). The deflection past the load point is given by

$$\begin{aligned} \delta ^*(X)=-\frac{F2_{(i=2)}.(L2M-{\mathfrak {T}})^{2}}{6.E.{\mathbb {I}}[X]}.((3.X-(L2M-{\mathfrak {T}}))) \end{aligned}$$

The same could be argued instead for just $F2_{(i=3)}$ applied applied $2.{\mathfrak {T}}$ short of the tip of the moveable digit and so on to $F2_{(i=18)}$ ﻿if appropriate.

Ignoring any load from the self-weight of the beam, some simple rules follow. Bending moment and curvature are zero distal of the (last) applied load. Bending moment and curvature increase towards the condyle. Bending (couple) moments and curvatures are additive.

**Table 5:** Tableau for moveable digit deformation ($\delta$) computation for the measurements of a single individual of a single UK astigmatan beehive species given the end of mastication surface $i_{e}=15$ ($\leftarrow$ indicates the extent of the occluding surface)

Location =	$x_{18}$	$x_{17}$	$x_{16}$	$x_{15}$	$\cdots$	$\cdots$	$x_{3}$	$x_{2}$	$x_{1}$
Feature =	Condyle			$i_{e}$	$\leftarrow$	$\leftarrow$	$\leftarrow$	$\leftarrow$	Tip
$X=$	$(L2M-17{\mathfrak {T}})$	${\mathfrak {T}}$	$2{\mathfrak {T}}$	$3{\mathfrak {T}}$	$\cdots$	$\cdots$	$(L2M-2{\mathfrak {T}})$	$(L2M-{\mathfrak {T}})$	*L*2*M*
${\mathbb {I}}[X]=$	$f(y^{*}_{18})$	$f(y^{*}_{17})$	$f(y^{*}_{16})$	$f(y^{*}_{15})$	$\cdots$	$\cdots$	$f(y^{*}_{3})$	$f(y^{*}_{2})$	$f(y^{*}_{1})$
$E[x]=$	1	1$\dag$	1$\dag$	1$\dag$	$\cdots$	$\cdots$	1$\dag$	1$\dag$	1$\dag$
$F2_{(i=1)}$	0	$\delta ({\mathfrak {T}})$	$\delta (2{\mathfrak {T}})$	$\delta (3{\mathfrak {T}})$	$\cdots$	$\cdots$	$\delta (L2M-2{\mathfrak {T}})$	$\delta (L2M-{\mathfrak {T}})$	$\delta (\varvec{L2M})$
$F2_{(i=2)}$	0	$\delta ({\mathfrak {T}})$	$\delta (2{\mathfrak {T}})$	$\delta (3{\mathfrak {T}})$	$\cdots$	$\cdots$	$\delta (L2M-2{\mathfrak {T}})$	$\delta (\varvec{L2M-{\mathfrak {T}}})$	*
$F2_{(i=3)}$	0	$\delta ({\mathfrak {T}})$	$\delta (2{\mathfrak {T}})$	$\delta (3{\mathfrak {T}})$	$\cdots$	$\cdots$	$\delta (\varvec{L2M-2{\mathfrak {T}}})$	*	*
$\vdots$	$\vdots$	$\vdots$	$\vdots$	$\vdots$	$\vdots$	$\vdots$	$\vdots$	$\vdots$	$\vdots$
$F2_{(i=i_{e})}$	0	$\delta ({\mathfrak {T}})$	$\delta (2{\mathfrak {T}})$	$\delta (\varvec{3{\mathfrak {T}}})$	*	$\cdots$	*	*	*
$F2_{(q>i_{e}})=\varvec{0}$	0	0	0	0	0	$\cdots$	0	0	0
$\delta$Profile =	0	$\sum _{col}$	$\sum _{col}$	$\sum _{col}$	$\sum _{col}$	$\cdots$	$\sum _{col}$	$\sum _{col}$	$\sum _{col}$

Note by definition for 18 projected locations ${\mathfrak {T}}=\frac{L2M}{17}$. * = $\delta ^{*}(X)$ extrapolated from last value (set to zero to omit that part of the filament if required). $\sum _{col}$ = sum of deflections in column between two solid lines for that location. *F*2 (equivalent load) values are fixed for each row (using distances in bold, see text). ${\mathbb {I}}(X)$ given as a function of adjusted *y* profile values (see text). $\dag$ = set to increasing powers of 1.48 (left to right) for text example. $\delta$Profile is summation of terms between double lines. Note $x_{i_{e}}$ can change over individuals and therefore where extrapolation occurs and where *F*2 is set to zero will change accordingly

**Fig. 20:**
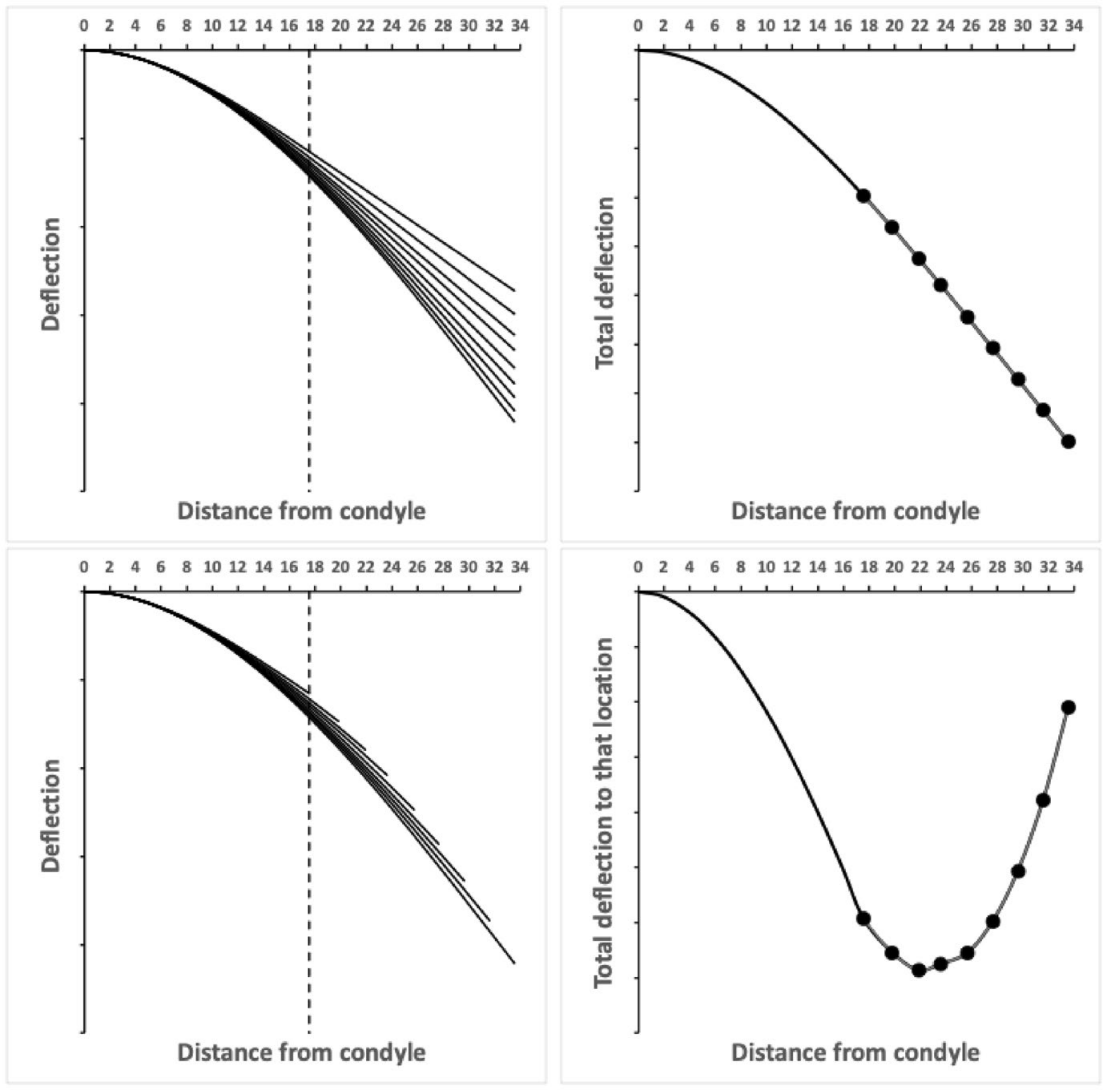
Example of unadorned moveable digit bending like a cantilever beam based upon *Glycyphagus domesticus* wild-collected specimen 224(2)-1 occlusive forces. Distance from condyle in $\mu$m. Upper. Left, deflection due to each adductive *F*2-derived load, each line extrapolated distally past its load point. Dashed line is end of mastication surface (at $x_{I_{e}}$). Only the load at the moveable digit tip induces a bend along the whole digit length. Right, total deflection, small black dots indicating zone where loads are applied during occlusion. Lower. Approximate model dropping extrapolated sections. *F*2 forces are accumulated as the focus moves proximally towards the condyle, the total deflection (at that point) of a beam comprising just those increasing number of ‘filaments’ increases to a maximum around just before the ascending ramus

So combining all these loads, Table 5 summarises the overall method focusing on the mastication surface data. However, the algebraic solution of a full system (Fig. 20 Upper) of hyperbolically changing applied forces (Bowman 2024) seemingly does not have a straightforward closed form. A numeric integration method would be needed that also allows for no forces proximal of the end of the mastication surface $x_{i_{e}}$, the actual dentition, the location of the neutral axis etc.

A simplification is called for. In some design sense the projecting part is neutral and might be ignorable evolutionarily (see below) so, an approximate artificial analogue to that full model (as follows) could be illuminating.

So firstly, put aside considering loads proximal to $x_{i_{e}}$ as there is no equal and opposite force generated here by the food stuff. Secondly, do the same calculation as above for each force $F2_{(i\ge 3...i_{e})}$ for the whole mastication surface (original $x=0...x_{i_{e}}$) i.e., with the new load points shifted by appropriate multiples of ${\mathfrak {T}}$ increments. Thirdly, abbreviate the moment arms at the point of the *F*2 load (as the digits slim down distally). Finally, then optimise over all the loads.

In doing this summation (in Table 5) over the loads, one is considering this like adding the deflections of several longitudinal ‘filaments’ sequentially recruited inside an overall composite beam (i.e., a ‘model system’ of the moveable digit whereby each filament of a here first uniform depth effectively takes, or compensates for, a different load over a different part of the tooth row). This simplification is a useful approximation to intuitively understand (without considering partial differential equations) and thus consider straightforwardly. From an evolutionary point of view, the extrapolated sections are completely determined by the design of their more proximal parts and so the summation could be legitimately dropped over those sections. Thus our model becomes as in Fig. 20 Lower.

Given that, the question arises - What single depth of each filament would obviate each and every filament bending and thus render a bar-like digit completely horizontal throughout?

**Fig. 21:**
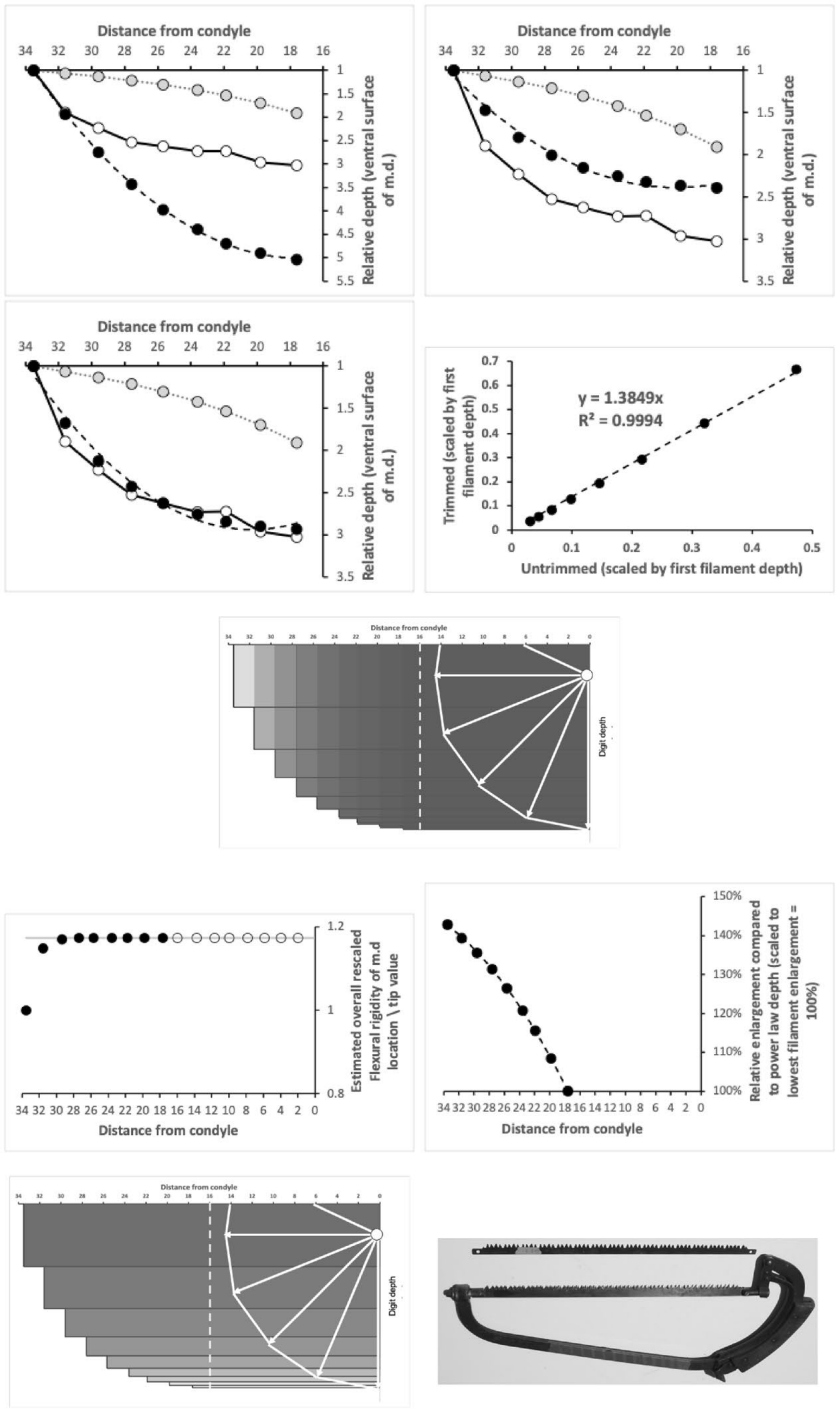
Reconstructed profiles based upon completely resisting occlusive forces based on *Glycyphagus domesticus* wild-collected specimen 224(2)-1. Distance from condyle in $\mu$m. Top row. Horizontal ramus ventral surface. Grey circles = $F2'$ profile Bowman (2023). Open circles = actual measured profile. Left, optimal solution = black circles with uniform Young’s modulus $E=1$ and same per ‘filament’ $y^{*}$ (i.e., fixed ${\mathbb {I}}[X]$ for all full length filaments). Right, optimal solution = black circles with $y^{*}$ (i.e., ${\mathbb {I}}[X]$) different for each ‘filament’ (but still $E=1$). Depth of full length filaments matches powers of 1.48 ($R^{2}=0.998$). Second row. Left, power law morphology but abbreviated filaments. Right, approximate linear relationship between full length and abbreviated filament depth estimates. Third row. Moveable digit abbreviated ‘filaments’ (layers) coloured by digit thickness at that point. Paler = less thick. Dashed white line = end of mastication surface at $x_{i_{e}}$. White wireframe is indicative of circular basal ramus at *L*1*U* units from condyle. Condyle = white circle with black outline. Fourth row. Left, flexural rigidity ($E.{\mathbb {I}}[X]$) by projected location (digit tip = 1) for ‘filament’ approximation of morphology. Right, extra ‘filament’ depth compared to power model (as an indicator of possible sclerotisation towards base-plate of dentition). Bottom row. Left. Graded shading showing possible non-linear sclerotisation by abbreviated filament. Darker = more likely. Right. A bow saw plus replacement blade (amended from StromBer 29 April 2008 under Creative Commons Attribution-Share Alike 2.0 Germany license from https://en.wikipedia.org/wiki/Polesaw)

To answer this, estimating the fixed $y^{*}$ depth by optimising a sums of squared deviation criterion (from the zero gradient of an unbent horizontal beam) can be done. For a fixed Young’s modulus of $E=1$ and ${\mathbb {I}}[X]=0.7854\ \forall \ X$ (i.e., $y^{*}=1$ for each load throughout), this gives Fig. 21 top row left.

However, although assuming a fixed constant filament depth (and full filament length) does produce a monotonically ventral profile, it does not give a realistic profile to the moveable digit ventral surface below the tooth row that matches actual measurements (Fig. 21, top row left).

How to improve this?

Now, if one allows the filaments to have different depths, heuristic search showed that a power series (close to powers of $\frac{3}{2}$) was the best simple function for the relative depth to obviate any digit deformation under the hyperbolically changing *F*2 values (note that linear, quadratic and exponential trends were also tried as alternatives).

One can sum all these deflection contributions (including extrapolates) separately over each projected location (i.e., over all the original $x_{i}$ values from 0....*L*2*M*), to yield a ‘static-loaded’ full total ‘deformation-resistive’ profile for the moveable digit (for it to be kept overall horizontal). These ‘strata’ compensating for the loads’ effects now match the ventral profile reasonably well if visually trimmed distally (see Fig. 21, top row right). However, this appears over-fitted. If one not just abbreviates the filaments visually but also leaves the extrapolated sections out of the calculations, one obtains a very good fit (Fig. 21 second row left). Note that the first filament depth remains the same, and the others scale approximately linearly (Fig. 21 second row right). If one colours each filament by its depth (given that $thickness=depth$ for a cylinder), this shows that the digit should be paler towards its tip (Fig. 21, third row). This resultant longitudinal and vertical pattern could be tested by quantitative assessment of the colour of digit regions on microscope slides in follow-up work. Of course, whether the digit is hollow or not, and the visual aspect of whether one is looking effectively edge on through the cuticle at the top and bottom of the digit, or transversely in the middle of the digit will need to be allowed for.

There is a small lack of fit (Fig. 21, second row left) around the distal parts of the moveable digit (i.e., the moment arms for the ‘upper’ filaments may need to be shorter than estimated). This is around $21\%$ drop off in flexural rigidity (Fig. 21, fourth row left), suggesting perhaps that pointed moveable digit tips are always sclerotised to compensate this, or alternatively perhaps are digit tips designed to be flexible and store some energy to be released on re-bound? Overall, this drop can either be obviated by proposing different values for Young’s modulus for each filament (i.e., strengthened micro-strata). Or, varying modulus values at any one location (anterior-posterior) within a filament (due to say micro-fibrils, local grains etc.,) are compensating for the loads’ effects. Either way this makes the rod’s overall resistance to bending *E* a function of the morphologically constructed *X* (i.e., $f(x_{i})$ within each species). Examining other mites (like oribatids) some of whom consume tough fibrous woody material could check for a strengthened digit tip (like a ceratopsian dinosaur beak) as well as the expected strong dentition.

Note that, given the assumed smooth morphological power law for the distally abbreviated filament depths, then the albeit small but indicated extra estimated depth for each filament (as a proportion of the most ventral filament increased depth) appears to be gently quadratic (Fig. 21, fourth row right) with filament abbreviation along the horizontal ramus. This suggests that using quantitative spectrometry to examine slide specimens for any progressive non-linear sclerotisation (increasing from the ventral surface of the moveable digit up to the *L*2*M* axis ‘base-plate’ zone allowing for thickness) as in the shading in Fig. 21 bottom row left might be useful in follow-up investigations.

A good validation of this model is that note how the ventral surface of the glycyphagid digit approximates a bow saw (Fig. 21 bottom row right).

Arthropod cuticle has a tensile strength in excess of aluminium and of a scale similar to that of bone (see Bowman 2024). Chitinous materials can show different Young’s moduli that vary in directions (Chen et al. 2022), but not majorly differ by arthropod origin (Caldwell and Mendez 2016). A strong stiffened base-plate (as in saws, Bowman (2024), effectively from an elevated Young’s modulus to resist dorsal elongation and buckling) should be needed for efficient dentition under time varying vertical loads during food mastication. Protein content in which the chitin fibrils are encased is important (Montroni et al. 2021), with sclerotisation dramatically increasing cuticular Young’s modulus by thousand folds (Vincent and Wegst 2004). Note that if the flexural rigidity $E.{\mathbb {I}}$ is fixed for a cylinder, a thousand fold increase in Young’s modulus can be adjusted for by a thousand fold decrease in ${\mathbb {I}}$, which is equivalent to around only a five fold increase in digit depth $y^{*}$ (as $\root 4 \of {1000}\approx 5.6$). Well within mite observed designs.

Indeed, then by inserting realistic ${\mathbb {I}}[X]$ relevant actual cross-sectional digit area values and a plausible pattern of Young’s moduli from digit observations, one could optimise over all these anisotropies (either for each wild-collected specimen separately with their unique end of mastication surface index $i_{e}$ values or over all individuals in that species) to produce profiles most like a horizontal line. This unknown positive function *E*[*x*] estimated ‘piece-wise’ over a grid could now be summarised across regions, compared across species using individualised estimated depths and common or individualised patterns in Young’s modulus or even related to the scale of other observations made on astigmatan digits such as optical density, measured coloration etc.

Considering acarine moveable digits as cantilevered beams is useful for comparative morphologists.

What does all this careful dissection of apparent complexity mean for the moveable digit as a whole?

**Fig. 22:**
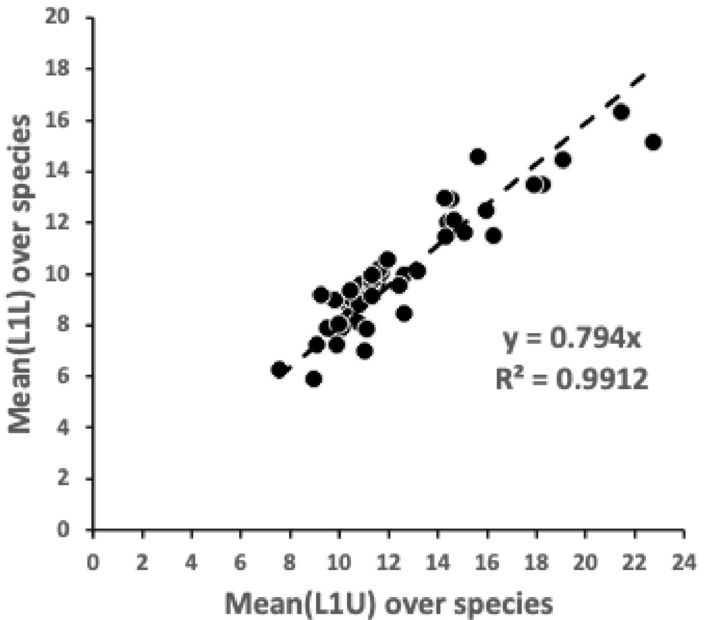
The basal ramus is broadly symmetric across the free-living Astigmata based upon analysis of data per species from Bowman (2021b). *L*1*U* = adductive (closing) chelal moment arm. *L*1*L* = abductive (opening) chelal moment arm. Black spot = each taxon of the 37 studied

- Firstly, the above gives an objective rationale for the use of empirical estimates derived from digit surface curvatures in mite ecomorphological studies (e.g., the perimeter measures in Liu et al. (2017); Adar et al. (2012)). Under a power law model, the accumulating depth of the digit (starting at the filament next to the *L*2*M* axis) over the digitising standard grid is given by a geometric series where, $total\ depth=\frac{a}{b^{0}}+\frac{a}{b^{1}}+\frac{a}{b^{2}}....=a.(1+\frac{1}{b^{1}}+\frac{1}{b^{2}}...)$ which for *n* filaments yields $total\ depth=a.(\frac{1-(\frac{1}{b})^{n}}{(1-\frac{1}{b})})$ for $b\ne 1$. Now, given an infinite number of filaments the continuum limit of this series converges to $total\ depth=\frac{a}{1-\frac{1}{b}}$ as $\frac{1}{b}<1$, making it easy to approximately summarise measured digit profiles empirically in terms of just two parameters for individual mites or species (on average). The fitted coefficient *a* indicating the depth of the likely dentition base-plate (that might be differentially sclerotised) and $\frac{1}{b}$ being the fitted ‘common ratio’ determining the profile curvedness. This then can be mapped linearly to the model system of abbreviated filaments. Such estimation could be done for either chelal digit - the moveable digit ventrally from tip to abductive (opening muscle) tendon, or the fixed digit dorsally from tip to dorsal lyrifissure.
- Secondly, the scheme above begs the question as to why the individual claws of oribatid mites (Pfingstl 2017; Pfingstl et al. 2020; Pfingstl and Kerschbaumer 2022; Kerschbaumer and Pfingstl 2023a, b) do not look like chelal digits proximally i.e. they are not reinforced more like isopod dactyli (Baillie et al. 2019; van der Wal and Haug 2020) near their articulations? Heethof and Koerner (2007) found that the soil living *Archegozetes longisetosus* could produce disproportionately high forces against both vertical (holding on) and horizontal (pulling) challenge which was related to substrate roughness. These workers proposed an ‘ice-skating’ mechanism for smooth surfaces whereby the oribatid’s claws scratch over the surface to find a position to hook into. Indeed, are oribatid claws then just passive hold-fasts that rely upon only their tips ‘grasping’ the substrate (perpendicularly to the claw axis) and the resultant forces (as they "...walk through not over the landscape...") being essentially longitudinal to the general axis like a coat-hook? This would match them with a spring-like function in obviating a tidal flow-rate dependant dislodgement parallel to the substrate for liminal species? In that sense inundated marine oribatids may not permanently ‘hang onto’ their substrate at all (like chelae grasp food) but rather just do not get easily dragged along by the water movement, their tarsal ‘hooks’ catching on irregularities in an episodic manner as they are buffeted. Consideration of the design and theory of mechanical fasteners (Jeffries and Lentink 2020) and biomimetic hooked anchoring devices (Saunders 2015a, b, 2016, Fiorello et al 2021) may be of use here. Gorb et al (2002) gives a useful calculation of the displacement of a hook at the point of the force application as a function of the applied force (F). More detailed follow-up observations in the wild (or in wave tanks with tribologically specified surfaces) and ‘tumbling’ individual oribatids are needed.
- Thirdly, the ascending+basal ramus (up to the tendon junction on the coronoid process) should approximate a smooth cornered square ($\approx L1U*L1U$) in 2D size given the above. Given optimal symmetry, this infers that the abductive opening chelal moment arm (*L*1*L*) to the tendon insertion should approximate *L*1*U*, the lack of needing high leverage to open the chela compensated by a trivial musculature (indeed only small musculature certainly would be needed for any basal ramus working like a counter-weight). Examination of oribatid specimens with synchrotron X-ray microtomography (Heethof and Norton 2009) confirms the reduced musculature. Analysis of data from Bowman (2021b) broadly supports the former (Fig. 22), although the tendons’ effective angle ($\alpha$) in Fig. 11 of Bowman (2024)) adjusted for both tendons (see Heethof and Norton 2009) will need to be taken into account and some foodstuffs may usefully need compaction down as the chela re-opens after a bite. Indeed the bulk modulus (a measure of a material’s decrease in volume with an increase in pressure on all sides) of mites’ typical foodstuffs in which they may burrow (Bowman 2023) needs assessment. Foods vary in their elastic properties over a wide range of behaviours (Povey and Hefft 2023). In the case of mastication, textures vary from hard and solid through brittle (e.g., chocolate bars) and crispy/crunchy (e.g., biscuits) to viscous and extensional flow (e.g., syrups including plant saps) and finally very low viscosity fluids (e.g., watery solutions). Mites should be tested against differing food toughness (as in primates Williams et al. 2005) in follow-up feeding trials. The reciprocal of the food’s bulk modulus (i.e., its ‘compressibility’) may be related to a mite’s chelal abductive force and thus to the mite’s opening musculature cross-sectional area. For sure, a moveable digit designed not to buckle or break under occlusive load should be fine for most food compaction needs (say when digging). Food compressibility (as well known in fishes, Mihalitsis and Bellwood (2017)) may also explain why acarine oesophagi sometimes at first seem far too small for grabbed and masticated material to get through.
- Finally, it lays the groundwork for comparative follow-up work using finite element analysis of acarine feeding mechanics as in theropods (Ma et al. 2002).

### How might the moveable digit patterns be explored further?

Hopefully, even if mathematics is not one’s forte, the reader has gained an intuitive appreciation of acarine moveable digit form and pattern from the previous sections. What next could be done? Firstly, variation in the transitional geodesic could be better estimated by collecting other reference species samples to yield more accurate empirical inferences. Furthermore, variation in the pattern matrix for *T. putrescentiae* could be explored by using a much finer digitising grid (to better capture distal profile features) and by collecting other samples from different locales and habitats for testing as this acarid appears to be a particularly variably formed species.

While culturing does appear to have affected the chelal morphology of *C. lactis*, it may not be a strong factor for other more generalist feeding (see Bowman 2021b) saprophagous astigmatan species. This is because if one excludes the moveable digit tip velocity ratio values for Ca4 (and the specialist durophage *Dermatophagoides microceras*, D5) from the results in Bowman (2021b) and plots the remainder against year of collection, then under an assumption of a random collection of species, no relationship to velocity ratio is found (*plot not shown*, $R^{2}=0.0237$). So, cultured or preserved specimens within a particular trophic functional group may still be useful for comparative morphologists (although recently collected individuals would always be ideal). What the species were eating when collected would be pertinent to record.

Secondly ("*...just for hardcore morphologists...*"), tangent planes formed at the consensus $\varOmega _{Clactis,j,k}$ and the consensus $\varOmega _{Gdomes,j,k}$ matrices may show different SVD decompositions at each point. That is their pattern (within *k*) may vary in strong specific ways. So, one could argue that estimating the typical *dsigma* in this paper is ignoring that. Although the minimum length geodesics from the test $\varOmega$ matrices to the $\varOmega$ interpolate matrices do intersect at a right angle on the curved surface, the direction of the distances *dClactis* and *dGdomesticus* defined by the within-species ‘jitter’ on the surface could be different. Indeed, the tangent planes formed at the consensus $\varOmega _{Clactis_{k}}$ and $\varOmega _{Gdomes_{k}}$ themselves could show different SVD decompositions at each point.

Accordingly, differences in structure could be explored by examining the main eigenvectors of the Riemannian measure i.e., $SVD(log[\varOmega _{a}^{-\frac{1}{2}}\varOmega _{b}\varOmega _{a}^{-\frac{1}{2}}])$ where *a*, *b* are any matrices of interest to be compared. This is particularly so in a follow-up study of other relatively small astigmatan species (e.g., those in and around T13 and G5 in Fig. 2).

*To be explicit for "the enthusiastic analyst"*, note that a square symmetric full rank diagonizable matrix like $\varOmega =[\varOmega _{a}^{-\frac{1}{2}}\varOmega _{b}\varOmega _{a}^{-\frac{1}{2}}]$ can be factorised to the form $\varOmega =Q.\varLambda . Q^{-1}$ where *Q* is a *p* by *p* square matrix whose *i*th column is the *i*th eigenvector of $\varOmega$ and $\varLambda$ is a diagonal matrix whose diagonal elements are the corresponding eigenvalues, i.e., $\varLambda _{i,i}=\lambda _{i}$. Eigenvalues should be positive unless the matrix is ill conditioned (and thus subject to computational difficulties). Note also that, *Q* formed by the $\varOmega$ eigenvectors here is guaranteed to be an orthogonal matrix, therefore $Q^{-1}=Q^{T}$.

Then the standard result (e.g., Dieci (1966)) applies that $log(\varOmega )=Q.log(\varLambda ).Q^{-1}$ which because $\varLambda$ is diagonal $\Rightarrow$

$$\begin{aligned} log(\varOmega )=Q.\left( \begin{array}{cccc} log(\lambda _{1}) &{} 0&{} \cdots &{} 0 \\ 0 &{} log(\lambda _{2}) &{}\cdots &{} 0\\ \vdots &{} \vdots &{} \ddots &{} \vdots \\ 0&{} 0 &{} 0 &{} log(\lambda _{p}) \end{array} \right) .Q^{T} \end{aligned}$$

In other words, the exploration of the Riemannian measure ‘components’ is by the relative eigenvectors (*Q*) from $SVD([\varOmega _{a}^{-\frac{1}{2}}\varOmega _{b}\varOmega _{a}^{-\frac{1}{2}}])$ rescaled by *log* their original eigenvalues.

Examining the large positive (*log*) and large negative (*log*) eigenvalue vectors will be enlightening in determining the overall relative directions (Bookstein 2018) of the profile’s transition. Then, since the eigenvalues of an inverse matrix are equal to the inverse of the eigenvalues (i.e., $\frac{1}{\lambda _{ii}}$) of the original matrix, the large positive *log* eigenvalues indicate the vectors of difference looking from the $\varOmega _{a}$ end of the reference geodesic and the large negative *log* eigenvalues the vectors of difference looking from the other $\varOmega _{b}$ end. The Riemannian distance between $\varOmega _{a}$ and $\varOmega _{b}$ is $\sqrt{(\sum _{i=1}^{p}log^{2}(\lambda _{i}))}$ (Barachant et al. 2013). It is a metric invariant under inversion and affine transformations (i.e., changes of co-ordinate system or transformations that preserve collinearity - in classical geometry, those of translation, rotation and reflection in an axis) Metric for Forstner and Moonen (2003).

**Fig. 23:**
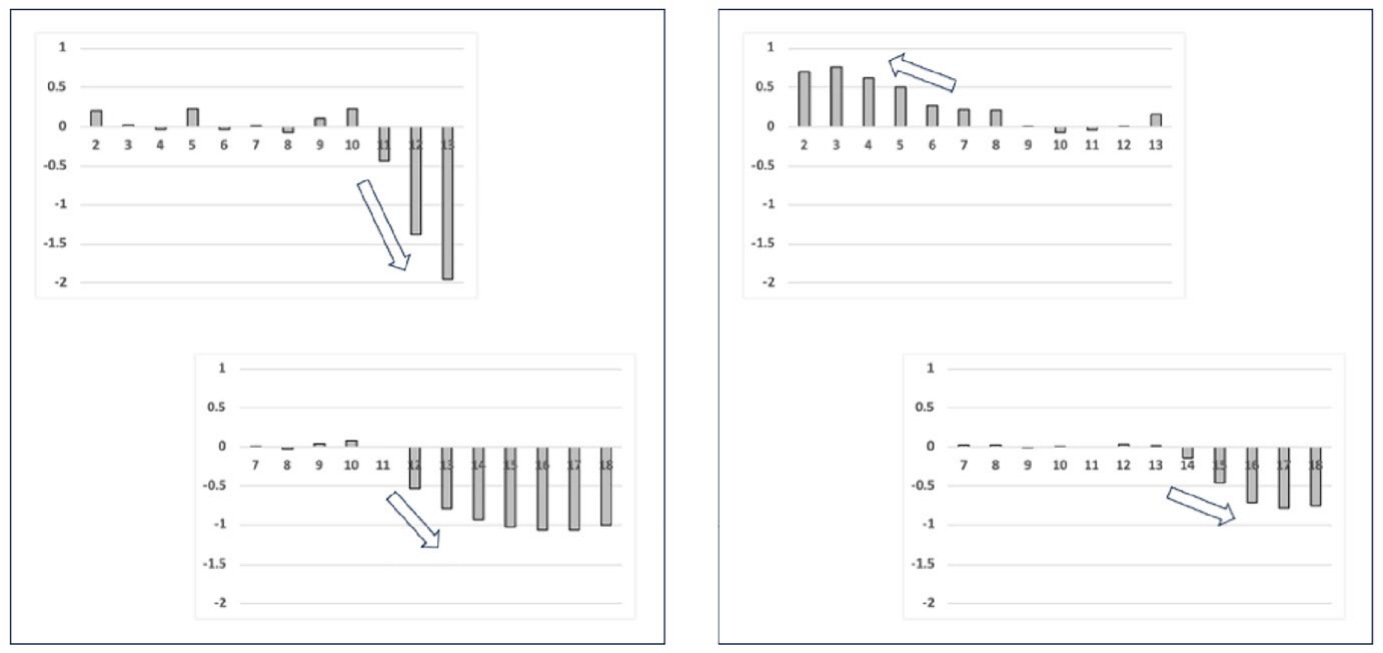
First eigen component loadings per item ( = projected location) calculated by Riemannian metric relative $eigenvector_{1}.\sqrt{log(eigenvalue_{1})}$. Left component coefficients from the consensus *Glycyphagus domesticus* point of view. Right from the consensus *Carpoglyphus lactis* point of view. Note how the scale of the component loading coefficients reflect the relative steepness of geodesic in Fig. 15. Subfigures are aligned vertically as distal and proximal regions, moveable digit tip to the left and condyle to the right. Large arrows indicate swelling (upwards), shrinking (downwards) as one moves away from that consensus position

Empirical plots of ordered *log*(*eigenvalues*) or the Grad method (Lue and Zhang 2000) can be used to judge the importance of each vector, recalling that eigenvalues represent the total amount of variance that can be explained by a given eigenvector and that eigenvalues close to zero (not logged) imply there is item multicollinearity. Zero eigenvalues suggest reduced dimensionality of the original data. Note that the eigenvector times the square root of the eigenvalue gives the ‘component loadings’ which can be interpreted as the correlation of each item with the principal component (Fig. 23).

This when plotted nicely shows agreement with geodesic steepness (Fig. 17) and the postulated evolutionary path in Fig. 16. Looking from *G. domesticus* towards *C. lactis*, the (relative) component loadings indicate a diminution in the adductive lever moment arm *L*1*U*, a declining basal ramus robustness and a particular lowering of the ascending ramus all consilient with the known velocity ratio and adductive force changes (see Bowman 2021b, 2023). Looking from *C. lactis* towards *G. domesticus*, the (relative) component loadings indicate a robustification of the bar-like digit distally and the commensurate gentle fall in the height of the basal ramus ready for the shortening of the digit’s mastication surface and the effective movement of the ascending ramus anteriorly (Fig. 16) before basal ramus swelling again. These loadings can also be used with the original projected locations data to optimally plot the data in various Euclidean dimensions. Directions which are mutually orthogonal (perpendicular) to all of the vectors in any *Q* can then be found in the ‘orthogonal complement’ of any *Q* matrix if required.

**Fig. 24:**
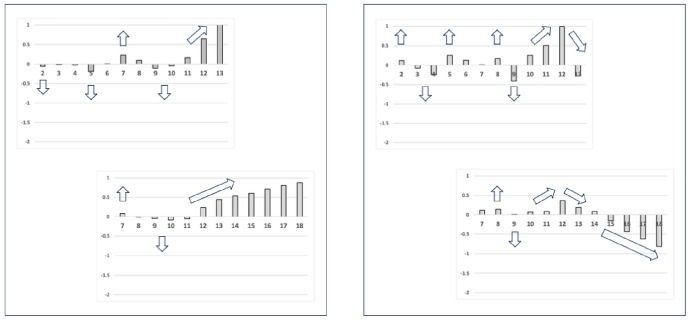
First eigen component loadings per item ( = projected location) calculated by Riemannian metric relative $eigenvector_{1}.\sqrt{log(eigenvalue_{1})}$. Left component coefficients from the consensus interpolate point of view. Right from the wild-collected *Tyrophagus putrescentiae* point of view. Note how the scale of the component loading coefficients reflect the locations with stars in Fig. 17. Subfigures are aligned vertically as distal and proximal regions, moveable digit tip to the left and condyle to the right. Large arrows indicate swelling (upwards), shrinking (downwards) as one moves away from that consensus position

Thirdly, examining in the same way as Fig. 23, the transition to and from the wild-collected *T. putrescentiae* profile and its nearest interpolate on the reference consensus geodesic gives Fig. 24. In this, one can see that moving away from the interpolate towards the wild-collected sample is in the direction of the development of particular heterodonty (especially around $x_{7}$), a systematic change in the ascending ramus ($x_{11}...x_{13}$) and basal ramus growth. Transition away from the wild-collected sample profile is dominated by the direction of basal ramus shrinkage, plus some changes in the ascending ramus shape and a complicated pattern of smoothing out the observed heterodonty.

Fourthly, it could be very illuminating for acarologists to assess whether *Dermatophagoides* spp. are heterodontous or not, and to conduct a selection experiment on many different pyroglyphid mites (including *Euroglyphus maynei*) by giving them different materials to eat for an extended period to see if their moveable digit form (as food specialists) is also evolutionarily plastic like in *C. lactis* (and in what detailed way it changes).

Fifthly, given that *T. putrescentiae* appears to be so plastic morphologically, selection experiments using a wide variety of foods could be very illuminating to understand its pest potential morphologically. The resultant multiple $\varOmega$ matrices could be summarised by a sample Fréchet mean (Pennec et al. 2006; Moakher and Zéraï 2011). How anisotropic this matrix is could be assessed using the method in Fletcher and Joshi (2007). Does character displacement occur in different communities featuring this cosmopolitan species?

Sixthly, the next level of complexity dissection could be taken - finite element analysis. This is deployed in understanding engineering structures and could be used to visualise acarine digit 3D stresses and strains like in insect mandibles (Klunk et al 2023) and dinosaurs (e.g., Barrett and Rayfiled (2006); Lautenschlager et al. (2013)). This would require detailed modelling of the variation in depth and thickness of digits to estimate its actual volumetrics in follow-up morphological work (rather than just relying on the simpler ‘wavy-edged’ cylindrical simplifications above).

Finally, there remains the challenge for acarologists to examine much larger morphologically differentiated astigmatans (shown in the top right of Fig. 2 above the transverse cut-off line). Indeed if *Dermatophagoides pteronyssinus* D3 was defined as the other reference taxon to *C. lactis* (rather than G5), would now G5 sit on that new transitional geodesic or not? Could this analytical approach be used with astigmatans from other nidicolous biomes like birds nests? For sure *G. domesticus* can be found there (Hughes 1976). Could cadavers (Braig and Perotti 2009) be considered as another comparative habitat? There is much for acarologists to explore.

### Extending the results

Again for *"...hardcore morphologists..."* the numerical methods can be extended.

First of all, one could examine the condition number of the $\varOmega$ matrices (i.e., their sensitivity to initial perturbation) using Frechet derivatives. Frechet regression could be used to relate differences in digit patterns to various cheliceral size measures.

Secondly one could try decomposing $\varOmega$ matrices into parts. For sure, the results herein indicate that the pattern of moveable digit features may be somewhat decoupled from known cheliceral and chelal adaptations (Bowman 2021b).

Indeed, $\varOmega$ is useful for comparative acarology as it encompasses both differences in the mean profiles between species as well as the variation around such. This is because, recall that for a single variate *y*, its population variance $\sigma ^{2}$ is the average sums of squared differences $\tfrac{1}{n}\cdot \sum _{i=1}^{n}(y_{i}-\mu )^{2}$ where $\mu$ is *mean*(*y*). This can be expressed as the scaled sums-of-squares $\tfrac{1}{n}\cdot \sum _{i=1}^{n}(y_{i}^{2}-n \cdot \mu ^{2})$ where the differences represent random (stochastic) errors. In this form it can be seen that $\sigma ^{2}=E[y^{2}]-(E[y])^{2}$ where *E*[...] is the expectation operator.

So, for a *p*-column vector of measured *y*-variables of *n* individual profiles (rows herein), the corresponding $p\ by\ p$ traditional covariance matrix is

$$\begin{aligned} \varGamma =\frac{1}{n}\left[ YY^{T}-n\cdot {\bar{Y}}{\bar{Y}}^{T}\right] \end{aligned}$$

where ${\bar{Y}}$ is the mean for each *y*-variate and ^T^ means matrix transpose. Now ${\bar{Y}}$ is the $1\ by\ p$ column means, so it can be written ${\bar{y}}$. This infers that

1 $$\begin{aligned} {\bar{y}}{\bar{y}}^{T}+\varGamma =\frac{1}{n}YY^{T}=\varOmega \end{aligned}$$

So, removing the mean profile part to examine the *pattern* of ‘errors‘ within $\varOmega$ and examining the $p\ by\ p$ $\varGamma$ matrix may thus be done i.e., $\varGamma =\varOmega -{\bar{y}}{\bar{y}}^{T}$

Now, $\varGamma$ is the classic (mean corrected sums of squares and cross-products) CSSCP matrix used by many zoologists. The average sums-of-squares-and-cross-products (*avSSCP*) matrix (i.e., the term on the righthand side of equation 1) thus comprises a term for the macro-scale mean profile (i.e., ${\bar{y}}{\bar{y}}^{T}$) plus a term ($\varGamma$) for the stochastic micro-scale variation around that mean profile. If $\varGamma$ is pursued then data could be easily simulated using the Wishart distribution (see Bookstein 2018) and ‘synthetic’ individual mite profiles illustrated.

Judging ${\bar{y}}{\bar{y}}^{T}$ needs to be in the context of what the arrangement along the moveable digit looks like quantitatively if there had been no evolutionary differentiation at all (i.e., where the moveable digit is assumed to be an unornamented bar-like beam of uniform density in form, itself subject to selection pressures at the whole cheliceral scale). For the horizontal ramus this was already explored in Bowman (2024) using *t*, $\chi ^2$ and *F*-tests of the actual observed versus expected moveable digit feature sizes, velocity ratios and their variances. In that review, tribologically the chelae of the three species were found to be distinct, their action matching different sorts of macro-scale human tools.

If required, $\varGamma$ can be standardised into an ‘intrinsic variation’ correlation matrix ($\varXi$) through appropriate division by variance terms (so that all measures are upon an equi-variance footing and more variable measures do not dominate any SVD decompositions) to yield a scaled CCSCP (correlation) matrix.

Does $\varGamma$ and $\varXi$ indeed confirm the partial decoupling of digit feature patterns from cheliceral and chelal adaptations and thus how the whole trophic composite tool system is rationally designed at every level of scale in astigmatan mites? This awaits follow-up work.

Similarly, as the poet Jonathan Swift in 1733 said in Poems II. 651: “Big fleas have little fleas upon their backs to bite them, and little fleas have lesser fleas, and so *ad infinitum*”. Are there in turn patterns of features even on single teeth themselves e.g., like the fine ridges on the back of pliosaur teeth which facilitate a quick extraction of its dagger-like fangs? This all awaits follow-up mite work by comparative morphologists.

Finally, other analysis approaches for the digit profile could be tried by acarologists. For instance, smoothed multi-scale and Fourier analysis (Cesar and Costa 1997; Lestrel 2008); well-known neural net Machine Learning methods used for reading handwritten digits (e.g., Islam et al. (2017)); the functional analysis of continuous curving data (see Srivastava and Klassen 2016 survey), the conversion to tangent-direction coordinates or curvature coordinates (Bookstein 1978; Marcus 1990) or perhaps special methods deployed by palaeoanthropologists and palaeontologists in the functional analysis of chewing facets in mammals, especially humans (Laird et al. 2016 is an entry point here). There are lots of other possibilities for acarologists to pursue.

## Conclusion

The micro-saprophagous acarid *Tyrophagus putrescentiae* does not have an intermediate pattern of trophic functional form between the other two studied species *Carpoglyphus lactis* (Carpoglyphidae) and *Glycyphagus domesticus* (Glycyphagidae).

**Fig. 25:**
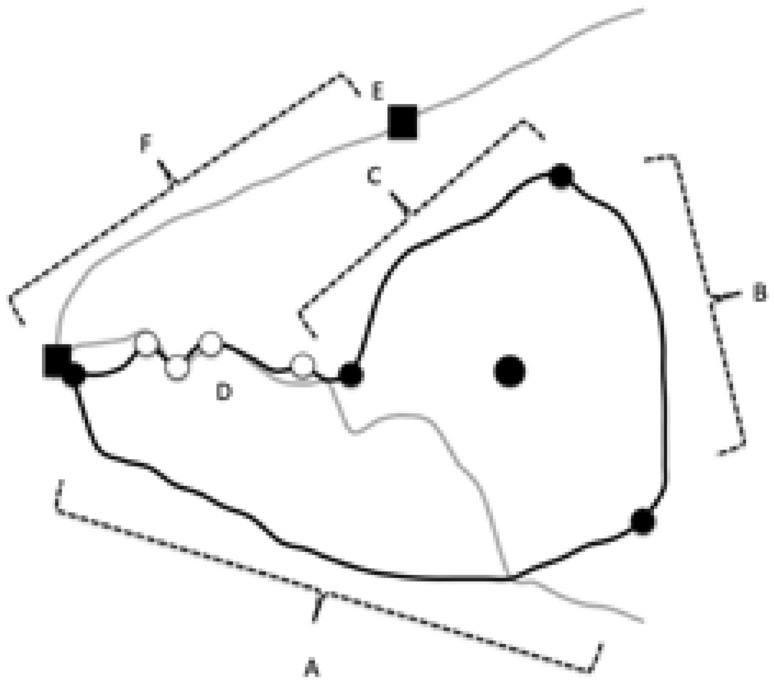
Suggested measurement scheme for future free-living acarine geometric morphometric studies. The posterior end of the tooth row (region D) and the curves A and F have been used in mesostigmatid studies. The open circles are randomly illustrated (see text for further explanation)

In the light of this study’s results, Fig. 25 outlines proposed landmarks and semi-landmarks for future mite studies.

The black circles indicate moveable digit homologous landmarks across species (including the posterior end of the tooth row at the end of the mastication surface). The square landmarks are those for the fixed digit (including ‘E’ for the dorsal lyrifissure), only to be simultaneously measured with the moveable digit locations on closed chelae. The open circles depend upon which species are included and are rationally chosen landmarks along the tooth row if the set of species have the same number of teeth or gullets across the investigated species. If this is not the case (and oribatids appear to have only have two teeth Heethof and Norton 2009) then choosing just the maximum tooth and the maximum gullet as two homologous landmarks across species may be useful to detect trophic changes. For sure, the teeth and gullets of *C. lactis* are not clear homologues with those of the other two species. Four regions of semi-landmarks are suggested for different reasons.

- Semi-landmarks along the lower moveable digit profile range A will help verify cheliceral height landmarks (as the depth of the moveable digit relies upon crushing force derived from the muscles within the cheliceral base, Bowman (2023)). This may be important as the cheliceral height can be biased due to slide compression.
- Semi-landmarks along the moveable digit profile range B will benchmark the general shape and overall size of the whole digit that bites into the foodstuff, characterise the basal ramus form and should help validate cheliceral size measures.
- Semi-landmarks along the moveable digit profile range C will help measure various kinds of subtle digit ascending ramus functional changes (especially when compared to the benchmark basal ramus form B) required for certain specialised feeding behaviours (and those which may arise from long-term eating of the ‘atypical food’). It can also highlight reinforcement of the adductive tendon junction.
- Semi-landmarks along range F will allow an investigator to look for underbite and overbite changes as found in mesostigmatids.

## Data Availability

No datasets were generated or analysed during the current study.

## Explanatory appendix

### Digit profile bending energy

Consider the moveable digit profile as a two-dimensional line function (of infinitesimal depth).

Using the Bernoulli-Euler theory of Euclidean elasticity (Landau and Lifshitz 1959) one can show that the 2D digit profile bending energy (Young et al. 1974) is directly proportional to the total squared curvature ($\kappa$) in an ideal deformed inextensible thin circular uniform cross-section rod (i.e., $BE \propto \int \kappa ^{2}ds$, see below). Such a rod is straight when unstressed (i.e., it has a reference configuration). Herein, no twisting is assumed (i.e., the bending is within the 2D plane). The more folded its surface is and the more sharper the folds, the higher is the bending energy. The circle is the shape which minimises the stored energy in a linear and thin-shelled medium. Indeed, the bending energy associated with a circle of radius $\rho$ is inversely proportional to its area. $\kappa$ as a shape parameter may be of use to evolutionary acarologists.

Following Palmer and Pámpano (2020), for a smooth regular plane curve *C* at a point *p* (on *C*) consider an infinitesimal arc of length *ds*. Replace this arc by the corresponding arc of an osculating circle to *C* at *p* i.e., a circle having the same curvature and tangent as *C* has at *p*. Define the radius of this circle as $\rho$ (the ‘radius of curvature’ of *C* at *p*). Under a displacement of the curve *C*, the radius will change (by *r*) and the arc length will also change. The consequent strain (i.e., the deformation or change in the shape of a material resulting from an applied force) is $\varUpsilon =\frac{r}{\rho }$. The corresponding stress (i.e., the force applied to the material per unit area) $\sigma =E.\varUpsilon =E.\frac{r}{\rho }$, where *E* is the Young’s modulus (which measures the rod’s resistance to bending i.e., it’s ‘stiffness’). Beware $\sigma$ here is not the $\sigma$ used elsewhere as a statistical variation measure, nor is *E* the expectation operator.

Let ${\mathfrak {B}}$ denote the cross-sectional surface of the rod, then the ($d\varUpsilon$) contribution to the potential energy of the curve is defined as

$$\begin{aligned} d\varUpsilon =\frac{1}{2}.\frac{E}{\rho }\left( \int _{{\mathfrak {B}}}r^{2}\ d{\mathfrak {B}}\right) ds=\frac{1}{2}E.\kappa ^{2}.{\mathbb {I}}\ ds \end{aligned}$$

from the infinitesimal arc (*ds*), where $\rho =\frac{1}{\kappa }$, $\kappa$ is the curvature ($\kappa >0$), and ${\mathbb {I}}$ is the second moment of area (or ‘moment of inertia’). The latter is a completely geometric measure (see below). So, the total potential energy of the deformed rod $\varUpsilon =\frac{1}{2}\int E.\kappa ^{2}.{\mathbb {I}}\ ds$

How to convert this to where *C* is defined in Cartesian co-ordinates, i.e., as $C=f([x,y])$ for an astigmatan moveable digit profile?

Following the approach of Bob McGinty (https://www.continuummechanics.org, return to the osculating circle. By first principles, $\rho .\theta =s$ where $\theta$ is the angle of the arc, or in infinitesimal differential form $\rho \ d\theta =ds$. Divide both sides of this by infinitesimal *dx* to give $\rho .\frac{d\theta }{dx}=\frac{ds}{dx}$

Now, how to change variables in this?

- Firstly by definition for an arc of a circle, $tan(\theta )=\frac{dy}{dx}$ or $\theta =arctan\left(\frac{dy}{dx}\right)$
  - Then, differentiate the latter with respect to *x* to give the standard result $\frac{d\theta }{dx}=\frac{\frac{d^{2}y}{dx^{2}}}{1+\left(\frac{dy}{dx}\right)^2}$
- Secondly, take the Euclidean distance $ds^{2}=dx^{2}+dy^{2}$ (i.e., the Pythagorean relation) and divide both sides by $dx^{2}$ to give $\left( \frac{ds}{dx}\right) ^{2}=1+\left( \frac{dy}{dx}\right) ^{2}$
  - Then, square root both sides to give $\frac{ds}{dx}=\sqrt{1+\left(\frac{dy}{dx}\right)^{2}}$
- Substitute these two results into $\rho .\frac{d\theta }{dx}=\frac{ds}{dx}$ to give

$$\begin{aligned} \rho .\frac{\frac{d^{2}y}{dx^{2}}}{1+\left(\frac{dy}{dx}\right)^2}=\sqrt{1+\left(\frac{dy}{dx}\right)^{2}} \end{aligned}$$

which on rearrangment yields

$$\begin{aligned} \frac{1}{\rho }=\kappa =\frac{\frac{d^{2}y}{dx^{2}}}{\left(1+(\frac{dy}{dx}\right)^{2})^{\frac{3}{2}}} \end{aligned}$$

$\kappa$ encapsulates the shape of the functional form in a measure of ‘bending rigidity’.

Assume *E* (the Young’s modulus for the lengthwise stretching of the chitinous material involved) is homogeneous and it is the same over all astigmatan chelae, and in the first instance ${\mathbb {I}}$ is also constant over species (thus both can be lost along with the half in any proportionality constant for $\varUpsilon$), then

$$\begin{aligned} BE=\int \kappa ^{2}ds=\int \left( \frac{\frac{d^{2}y}{dx^{2}}}{\left(1+\left(\frac{dy}{dx}\right)^{2}\right)^{\frac{3}{2}}}\right) ^{2}.\sqrt{1+\left(\frac{dy}{dx}\right)^{2}}\ dx \end{aligned}$$

as $ds=\sqrt{1+\left(\frac{dy}{dx}\right)^{2}}\ dx$ from above. Yielding (Levien 2008) the integral

$$\begin{aligned} BE= \int \frac{\left( \frac{d^{2}y}{dx^{2}}\right) ^{2}}{\left(1+\left(\frac{dy}{dx}\right)^{2}\right)^{\frac{5}{2}}}\ dx \end{aligned}$$

Note that defining the *x*-axis along the rod (aka the moveable digit) and if the *y* deflections, and more importantly the deformed slope $\frac{dy}{dx}$ are both small, then if $\frac{dy}{dx}<<1$ it can be neglected in the equation and one obtains the intuitive $\frac{1}{\rho }\approx \frac{d^{2}y}{dx^{2}}$ widely used by numeric analysts (i.e., bending energy is the second derivative squared).

The bending energy integral above may have closed form (but not for a hyperbolic function like a velocity ratio). Rather than using numeric integration, it can be approximated discretely from the data (over a region)

$$\begin{aligned} {\widehat{BE}}_{low-upp}=\frac{1}{(x_{high}-x_{low})}.\sum _{i=low}^{upp}\left( \frac{c_{i}^{2}}{\sqrt{(1+g_{i}^{2})^{5}}}\right) \end{aligned}$$

as in Bowman (2024). This can be used to quantify the morphological differences between profiles.

### Continuum mechanics of solid digits

Now consider the two dimensional moveable digit profile to have a depth.

Firstly, for clarity of exposition in this section, denote the arc length as *L* rather than *s* again following the approach of Bob McGinty (see above). Recall from above that when considering a line function, the arc length *s* (*L* here) is related to the radius of curvature by $s \Rightarrow L=\rho .\theta$ where $\theta$ is the angle.

Then, matters become more complicated when the ‘up-down’ thickness of a line (like an actual beam-like physical moveable digit) is considered. If the latter is bent, then different layers of it are stretched or compressed by different amounts. The ‘outer’ portion (where ‘outer’ is furthest away from the centre of the radius) is stretched, the ‘inner’ is compressed and everything in between is affected by different amounts. All are bent by the same angle $\theta$ but $\rho$ varies with the thickness, so *L* varies as well. Indeed there will be a layer (and a unique $\rho$ for the digit cross-section) where the ‘bent’ length is the same as the original length under no deformation ($L_{0}$). This defines the ‘neutral axis’ of the moveable digit from which conventionally ‘up-down’ (*Y*) measures of the beam are taken.

Secondly, now the radius of curvature at any ‘up-down’ *Y* is $(\rho -Y)$ and the final length (*L*) at any such *Y* is given by $L=(\rho -Y).\theta$. However $L_{0}=\rho .\theta$ so the (longitudinal along the length of the beam) strain $\varUpsilon =\frac{L-L_{0}}{L_{0}}=\frac{(\rho -Y).\theta -\rho .\theta }{\rho .\theta }=-\frac{Y}{\rho }$ meaning that the strain is zero at the neutral axis (i.e., $Y=0$) and linear away from it. Thick (‘up-down’) objects have large *Y*, thin ones small *Y* thus explaining why thick objects have more bending stiffness than thin objects.

Correspondingly the stress (by assuming that there are no lateral loads or stresses acting on the beam and that the digit is thin relative to its length) is axiomatically the classical uniaxial tensive $\sigma =-\frac{E.Y}{\rho }$

This stress field (effectively around the ‘ends’ of the beam - https://mechanicalc.com/reference/beam-analysis) yields a bending moment (or couple) *M* now on the cross-section (${\mathfrak {B}}$) of the digit (not on a 1D line) as $-\int _{{\mathfrak {B}}}Y.\sigma \ d{\mathfrak {B}}$ where $\sigma \ d{\mathfrak {B}}$ is the force and *Y* is the moment arm (i.e., distance). However from above, $\sigma =-\frac{E.Y}{\rho }$ so inserting this and rearranging gives

$$\begin{aligned} M=\frac{E}{\rho }\int _{{\mathfrak {B}}}Y^{2}\ d{\mathfrak {B}} \end{aligned}$$

The integral in this is, of course, ${\mathbb {I}}$ from above, so

$$\begin{aligned} M=\frac{E.{\mathbb {I}}}{\rho }=\kappa .E.{\mathbb {I}} \end{aligned}$$

where $\kappa$ is the curvature, *E* is the material’s Young’s modulus (i.e., the ratio of tensile stress to tensile strain, where stress is the amount of force applied per unit area and strain is extension per unit length) and ${\mathbb {I}}$ is the second moment of area (for that digit section). The equation infers that *M* and *E* are proportional to each other. The term $E.{\mathbb {I}}$ is known as the ‘flexural rigidity’. Note that by rearranging the equations for *M* and $\sigma$ in terms of their common element $\frac{E}{\rho }$ and equating the two, yields that the stress (i.e., $\sigma$ the force applied to the material per unit area) of the moveable digit is $=\frac{M.Y}{{\mathbb {I}}}$

As before, if the bending is small ($\frac{dy}{dx}<<1$) then $\rho \approx \frac{d^{2}y}{dx^{2}}$ and $M=E.{\mathbb {I}}.\frac{d^{2}y}{dx^{2}}$

Next, for simplicity of exposition, the beam representing the horizontal ramus, and the bigger beam representing the ascending ramus, are taken to be cylinders (Fig. 19) where their diameter (at locations $x_{i}$) varies as they progress (i.e., they are ‘wavy’ in *y* profile). Indeed, Bowman (2021b) shows that the width of digits (*thick*) is similar to their height (i.e., their depth). The two regions are also seamlessly joined and continuous with each other longitudinally.

Temporarily, let us assume that for a species *M* (the bending moment at the condyle) is constant (i.e., set at an ‘acceptable design level’ evolutionarily for that species’ trophic habit), then *within a species* $E=f(\kappa .{\mathbb {I}})$ at any one location. Now,

- For a circular cross-section beam of diameter $D_{i}$ (at *L*2*M* axis location $x_{i}$), ${\mathbb {I}}_{i}=\frac{\pi .D{i}^{4}}{64}$ (https://www.doitpoms.ac.uk/tlplib/beam_bending/second_moment.php).
- For the moveable digit, $\frac{D_{i}}{2}=Y_{i}\approx y_{i}^{*}$. Set this as the measured profile $y_{i}$ adjusted by subtracting $1.5*min(y)$ over $i=1...18$ first, to ensure a positive physical depth to the ramus being taken to be the physical radius of the cylindrical digit at $x_{i}$. So $I_{i}=\frac{\pi }{4}.(y_{i}^{*})^{4}\Rightarrow \frac{1}{I_{i}}\propto \frac{1}{(y_{i}^{*})^{4}}$. This does arbitrarily infer that the neutral axis is at $1.5*min(y)$ i.e., half as much again from the maximum gullet with respect to the *L*2*M* axis, but it does ensure an effective line approximation for *s* at this gullet and some thickness at the digit tip (as in reality). Comparison to chelal illustrations in Bowman (2024) shows this to be visually reasonable.
- For $\kappa _{i}$ this is set to be $=\sqrt{BE_{i}}$, the square root of the contribution to the (line profile) bending energy at the *i*th location from the discrete approximation of the integral in the previous section above (i.e., $\frac{c_{i}^{2}}{\sqrt{(1+g_{i}^{2})^{5}}}$). This assumes that the energy stored is concentrated in the uppermost ‘base-plate’ part of the digit.

So given this, $E_{i}$ can be estimated over individuals for each astigmatan species and moveable digit region,

$$\begin{aligned} \widehat{E_{region}}\propto \frac{1}{region\ length}\sum _{i=low}^{upp}\frac{(y_{i}^{*})^{4}}{\sqrt{BE_{i}}} \end{aligned}$$

Region length given the scaling of profiles to a standard equi-spaced grid is acting like the number of elements in the summation. In this estimation if $BE_{I}=0$ then it was conservatively reset as the smallest positive value over the other locations.
